# Alpha kinase 3 signaling at the M-band maintains sarcomere integrity and proteostasis in striated muscle

**DOI:** 10.1038/s44161-023-00219-9

**Published:** 2023-02-15

**Authors:** James W. McNamara, Benjamin L. Parker, Holly K. Voges, Neda R. Mehdiabadi, Francesca Bolk, Feroz Ahmad, Jin D. Chung, Natalie Charitakis, Jeffrey Molendijk, Antonia T. L. Zech, Sean Lal, Mirana Ramialison, Kathy Karavendzas, Hayley L. Pointer, Petros Syrris, Luis R. Lopes, Perry M. Elliott, Gordon S. Lynch, Richard J. Mills, James E. Hudson, Kevin I. Watt, Enzo R. Porrello, David A. Elliott

**Affiliations:** 1 Murdoch Children’s Research Institute, Royal Children’s Hospital, Melbourne, Victoria Australia; 2 Melbourne Centre for Cardiovascular Genomics and Regenerative Medicine, Royal Children’s Hospital, Melbourne, Victoria Australia; 3 Department of Anatomy and Physiology, University of Melbourne, Melbourne, Victoria Australia; 4 Centre for Muscle Research, University of Melbourne, Melbourne, Victoria Australia; 5 Novo Nordisk Foundation Centre for Stem Cell Medicine (reNEW), Murdoch Children’s Research Institute, Melbourne, Victoria Australia; 6 School of Biomedical Sciences and Department of Paediatrics, University of Melbourne, Melbourne, Victoria Australia; 7 Precision Cardiovascular Laboratory, The University of Sydney, Sydney, New South Wales Australia; 8 Australian Regenerative Medicine Institute, Monash University, Melbourne, Victoria Australia; 9 Centre for Heart Muscle Disease, Institute of Cardiovascular Science, University College London, London, UK; 10 Barts Heart Centre, St. Bartholomew’s Hospital, Barts Health NHS Trust, London, UK; 11 Queensland Institute of Medical Research Berghofer Medical Research Institute, Brisbane, Queensland Australia; 12 Present Address: Australian Regenerative Medicine Institute, Monash University, Melbourne, Victoria Australia; 13 Present Address: Novo Nordisk Foundation Centre for Stem Cell Medicine (reNEW), Murdoch Children’s Research Institute, Melbourne, Victoria Australia

**Keywords:** Cell biology, Stem-cell differentiation, Cell signalling, Phosphorylation, Cardiovascular biology

## Abstract

Muscle contraction is driven by the molecular machinery of the sarcomere. As phosphorylation is a critical regulator of muscle function, the identification of regulatory kinases is important for understanding sarcomere biology. Pathogenic variants in alpha kinase 3 (*ALPK3*) cause cardiomyopathy and musculoskeletal disease, but little is known about this atypical kinase. Here we show that ALPK3 is an essential component of the M-band of the sarcomere and define the ALPK3-dependent phosphoproteome. ALPK3 deficiency impaired contractility both in human cardiac organoids and in the hearts of mice harboring a pathogenic truncating *Alpk3* variant. ALPK3-dependent phosphopeptides were enriched for sarcomeric components of the M-band and the ubiquitin-binding protein sequestosome-1 (SQSTM1) (also known as p62). Analysis of the ALPK3 interactome confirmed binding to M-band proteins including SQSTM1. In human pluripotent stem cell-derived cardiomyocytes modeling cardiomyopathic *ALPK3* mutations, sarcomeric organization and M-band localization of SQSTM1 were abnormal suggesting that this mechanism may underly disease pathogenesis.

## Abstract (web summary)

McNamara et al. show that ALPK3 is an M-band protein required for phosphorylation of sarcomeric proteins and components of the protein quality control apparatus. Further, the autophagy receptor sequestosome-1 requires ALPK3 activity for sarcomeric localization.

## Main

Hypertrophic cardiomyopathy (HCM) is defined as the abnormal thickening of the left ventricular wall, affecting an estimated 1 in 200 individuals^1,2^, making HCM the most common inherited cardiac disorder. Pathogenic variants in genes encoding contractile proteins of the sarcomere are the most prevalent genetic cause of HCM^2,3^. Critically, maintaining sarcomere integrity relies on quality control mechanisms that identify and remove components damaged under high mechanical and biochemical stress during muscle contraction^4–6^. The mechanisms by which cardiomyocytes (CMs) maintain sarcomere integrity are poorly understood. A key mechanosensory mechanism linking the sarcomere to protein quality control pathways is via the kinase domain of the giant sarcomeric protein titin, which recruits the ubiquitin-binding protein p62/sequestosome-1 (SQSTM1) to the M-band of the sarcomere^7,8^. Mutations in the titin kinase domain result in dislocation of SQSTM1 from the sarcomere into cytosolic aggregates. Very little is known about how sarcomeric signaling cascades at the M-band coordinate protein quality control pathways to maintain sarcomere integrity in striated muscle cells. Although coordinated phosphorylation of sarcomeric proteins has long been recognized as integral to cardiac contractility^9^, few M-band kinases have been identified but include muscle creatine kinase, phosphofructokinase and the titin kinase domain^7,10–12^. Therefore, defining the sarcomeric kinome and mode of action of kinases is important to provide insights into muscle function and cardiomyopathies.

Multiple genetic studies have linked variants in alpha kinase 3 (*ALPK3*) with HCM^13–17^. Furthermore, induced pluripotent stem cell (iPSC)-derived CMs from patients homozygous for *ALPK3* loss-of-function variants recapitulate aspects of the HCM phenotype^17^ and *Alpk3* knockout mice develop cardiomyopathy^18^. ALPK3 is a member of the atypical alpha kinase family, which have low homology to conventional kinases and are defined by the ability to phosphorylate residues within α-helices^19^. In immortalized cell lines, exogenously delivered ALPK3 localizes to the nucleus^20^; thus, it has been proposed to regulate transcription factors^13^. Recent stem cell-based modeling of ALPK3 suggested that ALPK3 is a sarcomere- and nucleus-localized pseudokinase^21^. However, the kinase domain of ALPK3 is highly similar to myosin heavy chain kinase, which phosphorylates the tail of myosin heavy chain in *Dictyostelium* to regulate cytoskeletal dynamics^22^. Given the homology to myosin heavy chain kinase, we hypothesized that ALPK3 may have a similar role in cytoskeletal signaling in the heart. Clinical data and cellular and animal models all demonstrate that ALPK3 signaling is critical for cardiac function; therefore, identifying the network of cardiac proteins that rely on ALPK3 activity may provide insights into the intracellular signaling mechanisms used to control cardiac contractility and, in turn, HCM disease progression, while suggesting new therapeutic targets.

In this study, we addressed the hypothesis that ALPK3 acts as a cytoskeletal kinase, by using *ALPK3* reporter and mutant human pluripotent stem cell (hPSC) lines, as well as mass spectrometry, to define the role of ALPK3 in muscle contraction and signaling. Our data demonstrate that ALPK3 localizes to the M-band of the sarcomere, which is a key regulatory node of sarcomere function^7^. We demonstrate that ALPK3 is required to maintain a functional contractile apparatus in both hPSC-derived and mouse CMs. Furthermore, phosphoproteomic analysis revealed that ALPK3 is required to maintain phosphorylation of key sarcomeric proteins. Co-immunoprecipitation (co-IP) experiments showed that ALPK3 binds to known M-band components such as obscurin (OBSCN). Finally, ALPK3 phosphorylates and is required for the sarcomeric localization of SQSTM1, an important transporter of polyubiquitinated proteins^23,24^. These findings suggest that ALPK3 is an important component of the signaling network that maintains functional sarcomeres.

## Results

### ALPK3 is a myogenic kinase at the M-band of the sarcomere

**Fig. 1:**
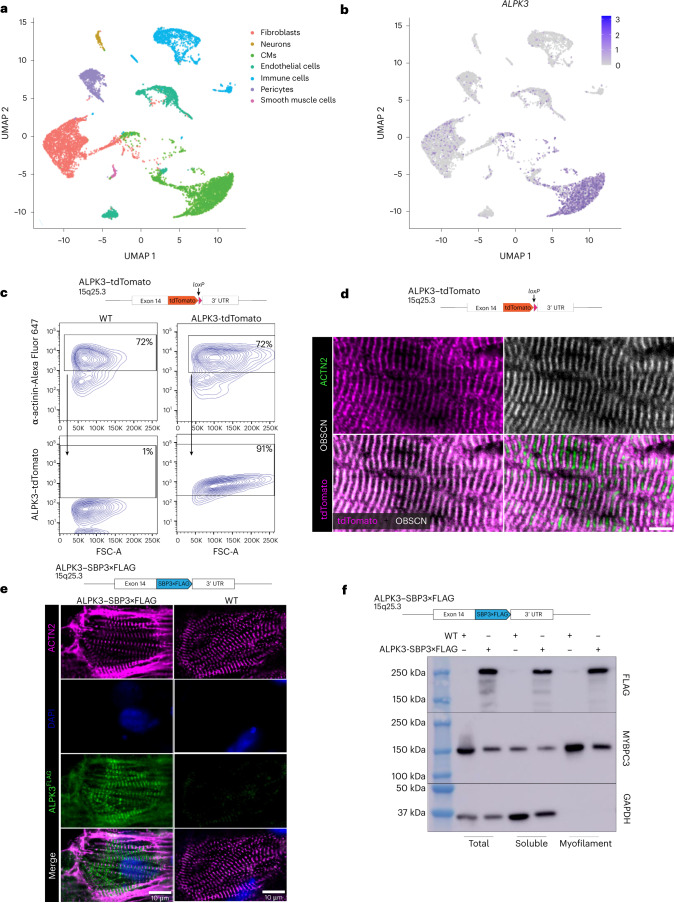
ALPK3 is a myogenic kinase localized to the M-band of the sarcomere. **a**, Uniform manifold approximation and projection (UMAP) plot generated from the snRNA-seq of three non-failing adult human hearts. **b**, Expression pattern of *ALPK3* in cell types of the non-failing adult human heart (colour key shows normalized log(expression) values). **c**, Schematic of targeting strategy of the ALPK3–tdTomato hPSC cell line and flow cytometry of directed cardiac differentiation for α-actinin and ALPK3–tdTomato. FSC, forward scatter. **d**, Representative immunofluorescence microscopy image of nanopatterned ALPK3–tdTomato hPSC-derived CMs stained for ALPK3–tdTomato (green), OBSCN (gray) and α-actinin (magenta); scale bar, 5 μm. **e**, Schematic of targeting strategy of the ALPK3–SBP3×FLAG hPSC cell line and representative immunofluorescence micrograph of WT and ALPK3–SBP3×FLAG hPSC CMs stained for α-actinin (magenta), FLAG (green) and DAPI (blue); scale bar, 10 μm. **f**, Schematic of targeting strategy of ALPK3–SBP3×FLAG hPSC cell line and western blot of ALPK3 in myofilament and cytosolic fractions of CMs. Source data

**Extended Data Fig. 1:**
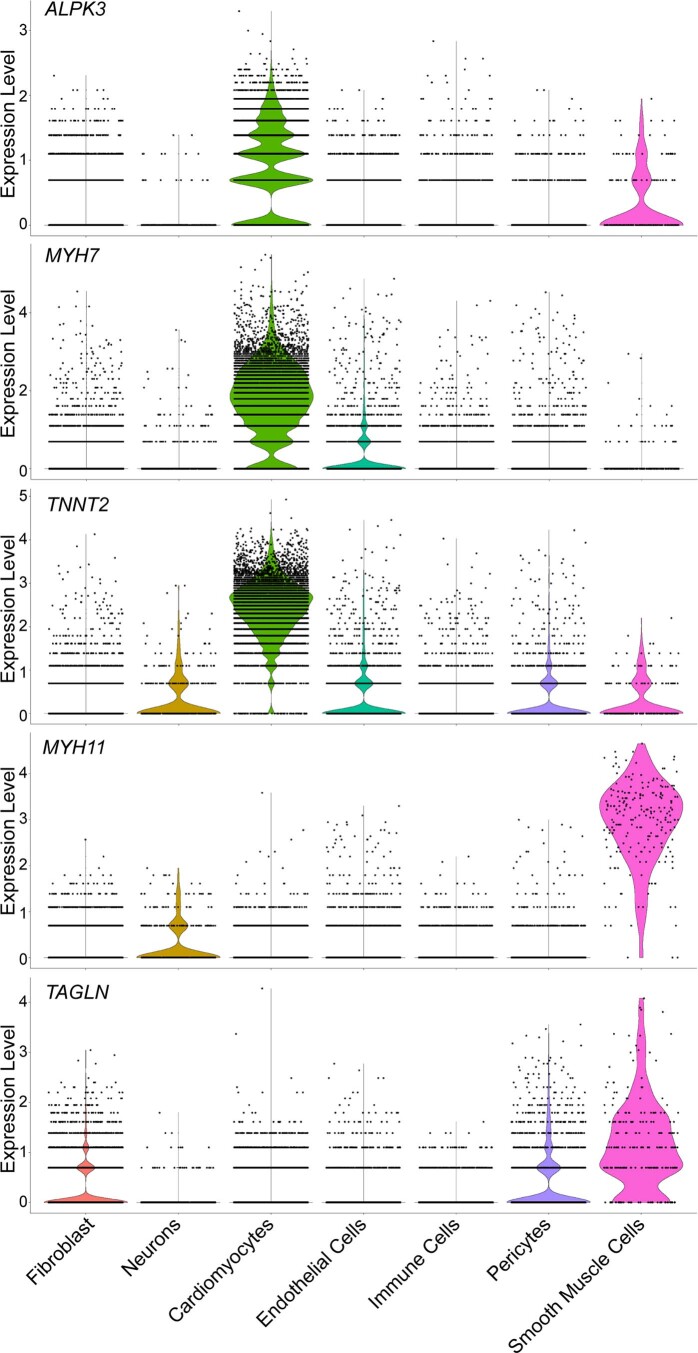
Single nucleus RNA sequencing. Violin plot of *ALPK3*, cardiomyocyte markers *MYH7 and TNNT2*, and smooth muscle cell markers *MYH11* and *TAGLN*. n = 3 adult hearts.

**Extended Data Fig. 2:**
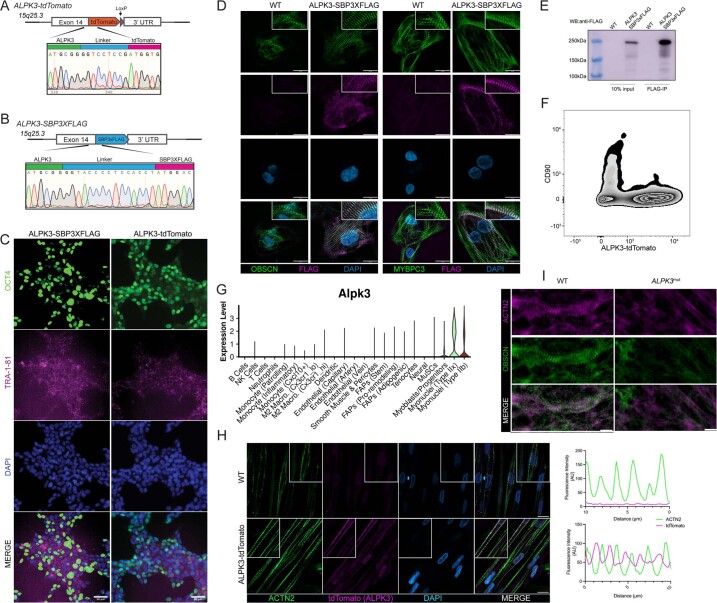
Validation of C-terminal tagged *ALPK3* hPSC cell lines. **a**. Gene targeting outline of *ALPK3-tdTomato* hPSC cell line and Sanger sequencing confirmation of junction. **b**. Gene targeting outline of *ALPK3-SBP3xFLAG* hPSC cell line and Sanger sequencing confirmation of junction of gene fusion. **c**. Stem cell markers of *ALPK3-SBP3xFLAG* and *ALPK3-tdTomato* hPSC cell lines. Scale bar = 50 µm. **d**. Representative immunofluorescent micrograph of *ALPK3-SBP3xFLAG* and WT hPSC-CM’s stained for OBSCN (green), FLAG (magenta), and DAPI (blue) or MYBPC3 (green), FLAG (magenta), and DAPI (blue). Scale bar = 20 µm. **e**. Western blot of WT and *ALPK3-SBP3xFLAG* hPSC-CMs immunoprecipitated with anti-FLAG antibody. **f**. Flow cytometry of *ALPK3-tdTomato* stained for stromal cell marker CD90. **g**. Violin plot of *ALPK3* expression across 23 cell types identified in mouse skeletal muscle. **h**. Representative immunofluorescent micrograph of WT and *ALPK3-tdTomato* hPSC-skeletal muscle stained for alpha-actinin (green), tdTomato (magenta), and DAPI. Scale bar = 15 µm. Line profile demonstrating alpha-actinin and ALPK3tdTomato organization. **i**. Immunofluorescence of WT and ALPK3mut hCOs staining alpha-actinin (magenta) and obscurin (green), scale bar = 10 µm. Source data

To define the expression of *ALPK3* in the human heart, we interrogated a single-nucleus RNA sequencing (snRNA-seq) dataset^25^ of non-failing adult left ventricle tissue (Fig. 1a). *ALPK3* transcripts were enriched within CMs (fold enrichment approximately 2.43, *P*_adj_ < 10^−100^), but also to a lesser extent in smooth muscle cells (fold enrichment approximately 1.21, *P*_adj_ = 6.63 × 10^−5^) (Fig. 1b and Extended Data Fig. 1). To determine the subcellular localization of ALPK3, we generated a series of *ALPK3* reporter hPSC lines in which either the tdTomato fluorescent protein or a streptavidin-binding peptide 3 (SBP3)×FLAG tag (SBP3×FLAG) was fused to the C terminus of endogenous ALPK3 (Extended Data Fig. 2a–c). ALPK3–tdTomato was strongly expressed in α-actinin-expressing hPSC-derived CMs but absent in CD90-expressing stromal cells (Fig. 1c and Extended Data Fig. 2f). Critically, both the ALPK3–tdTomato and ALPK3–SBP3×FLAG fusion proteins localized to the M-band of the sarcomere (Fig. 1d,e, Extended Data Fig. 2d and Supplementary Video 1) as demonstrated by interdigitation with Z-disc α-actinin-2 (ACTN2), colocalization with OBSCN and its position between the two A-bands of the sarcomere (MYBPC3). In three-dimensional (3D) reconstructions of live ALPK3–tdTomato hPSC CMs, ALPK3 formed striated patterns consistent with sarcomere localization; no nuclear localization was observed (Supplementary Videos 2 and 3). Moreover, cellular fractionation studies using CMs derived from the ALPK3–SBP3×FLAG (Extended Data Fig. 2b–e) line revealed that ALPK3 was associated with both the myofilament and cytosolic protein fraction (Fig. 1f). The clinical phenotype of *ALPK3* mutations also includes musculoskeletal defects^13,15,17^. Using a publicly available snRNA-seq dataset for mouse skeletal muscle, we found that *Alpk3* expression in skeletal muscle^26^ is also restricted largely to myofibers (Extended Data Fig. 2g). In iPSC-derived skeletal muscle cultures, ALPK3–tdTomato localized to the M-band, as demonstrated by its localization between Z-disc marker α-actinin (Extended Data Fig. 2h). Together, these results demonstrate that ALPK3 is restricted to myocytes and probably functions at the sarcomeric M-band in striated muscle. Our finding of ALPK3 at the contractile apparatus of myocytes challenges the current dogma that ALPK3 is a nuclear-localized^21^ regulator of transcription factors^13,20^. These data suggest an alternative hypothesis that ALPK3 is a regulatory kinase controlling cardiac contraction via phosphorylation of sarcomeric proteins.

### Sarcomere organization and calcium handling requires ALPK3

**Fig. 2:**
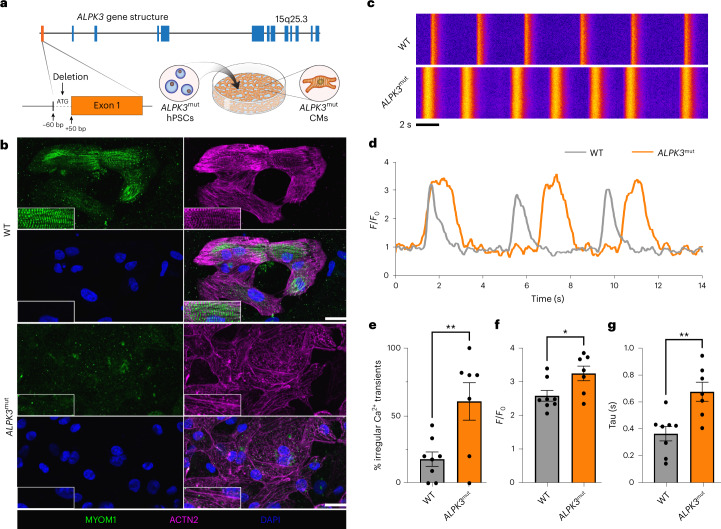
ALPK3 is required for sarcomere organization and calcium handling. **a**, *ALPK3* gene structure, schematic of *ALPK3*^mut^ gene targeting and graphic of cardiac differentiation. **b**, Representative immunofluorescence micrograph of WT and *ALPK3*^mut^ hPSC CMs stained for MYOM1 (green), α-actinin (purple) and DAPI (blue); scale bar, 30 μm. **c**, Representative line scans of Fluo-4 AM calcium handling in WT and *ALPK3*^mut^ hPSC CMs. **d**, Representative calcium transient trace of WT and *ALPK3*^mut^ hPSC CMs. **e**, Percentage of irregular calcium transients in WT and *ALPK3*^mut^ hPSC CMs. **P = 0.01. **f**, Peak systolic Fluo-4 AM fluorescence for WT and *ALPK3*^mut^ hPSC CMs. *P = 0.026. **g**, Time constant (tau) of diastolic calcium reuptake in WT and *ALPK3*^mut^ hPSC CMs. **P = 0.003. For the calcium handling data (**e**–**g**), *n* = 80 and 74 cardiomyocytes for WT and *ALPK3*^mut^ over eight and seven independent replicates, respectively. Data are shown as the mean ± s.e.m. Two-tailed Student’s *t*-test. Source data

**Extended Data Fig. 3:**
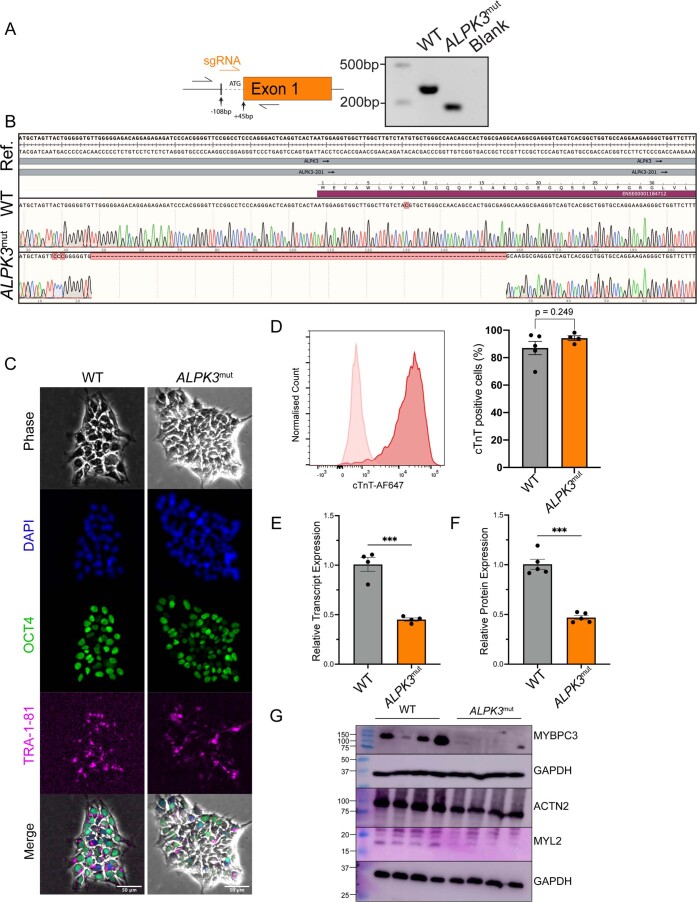
Validation of *ALPK3*^mut^ hPSC cell line. **a**. Gene targeting strategy with screening primers (black) and gRNA location (orange). PCR genotyping gel. **b** Sanger sequencing chromatograms of WT and *ALPK3*^mut^ amplicons against reference human genome. **c** Stem cell markers of WT and *ALPK3*^mut^ hPSC cell lines. Scale bar = 50 µm. **d** Flow cytometry of WT and *ALPK3*^mut^ hPSC-CMs using cardiac troponin-T. n = 5 for WT and 4 for *ALPK3*^mut^. Data is presented as mean ± S.E.M. Two-sided t-test.**e** Relative *ALPK3* RNAseq transcript levels by for day 30 purified WT and *ALPK3*^mut^ hPSC-CMs. n = 4 biological replicates. *** indicates p < 0.001 from two-sided t-test. Data is presented as mean ± S.E.M. **f** Relative ALPK3 protein levels by mass spectrometry for day 30 purified WT and *ALPK3*^mut^ hPSC-CMs. n = 5 biological replicates. *** indicates p < 0.001 from two-sided t-test. Data is presented as mean ± S.E.M. **g** Western blot of select sarcomeric proteins between day 30 purified WT and *ALPK3*^mut^ hPSC-CMs. Source data

To assess the regulatory role of ALPK3 in CM function, we used an *ALPK3* loss-of-function mutant (*ALPK3*^*c.1-60_+50del110*^, hereafter *ALPK3*^mut^) hPSC cell line (Fig. 2a and Extended Data Fig. 3a–c)^17^. Cardiac differentiation was unaffected in the *ALPK3*^mut^ line, which produced a similar proportion of hPSC-derived CMs to wild-type (WT) cells, indicated by cardiac troponin T-expressing cells (Extended Data Fig. 3d). These findings suggest that, unlike its paralog ALPK2 (ref. ^27^), ALPK3 is not required for cardiogenesis. *ALPK3*^mut^ CMs displayed disorganization of the sarcomeres and loss of the M-band protein myomesin (MYOM1). We also observed the presence of α-actinin-containing stress fiber-like structures and aggregates (Fig. 2b). Calcium transients in single CMs (Fig. 2c,d) recapitulated patient arrhythmogenicity in *ALPK3*^mut^ hPSC CMs^13,17^(Fig. 2e). Peak cytosolic calcium levels (Fig. 2f) were elevated while calcium reuptake was delayed in *ALPK3*^mut^ CMs (Fig. 2g). These results demonstrate that *ALPK3*^mut^ hPSC CMs recapitulate key hallmarks of human *ALPK3*-induced cardiomyopathy and suggest that ALPK3 has a key role in maintaining sarcomere integrity.

### ALPK3 deficiency impairs contractility

**Fig. 3:**
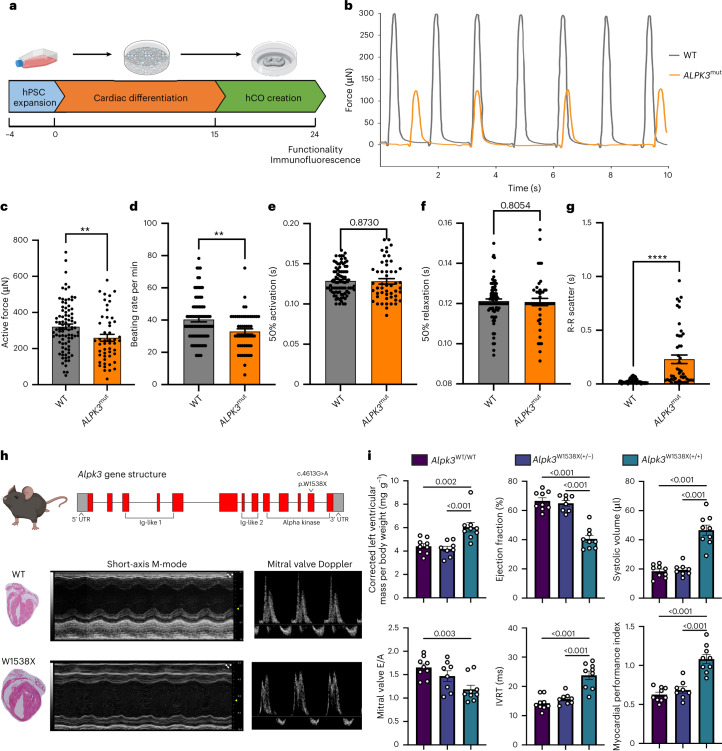
ALPK3 deficiency impairs contractility in hCOs. **a**, Experimental outline of the hCO study. **b**, Force traces from representative WT and *ALPK3*^mut^ hCOs. **c**, Total active force production from WT and *ALPK3*^mut^ hCOs. **P = 0.0073. **d**, WT and *ALPK3*^mut^ hCO beating rate per minute. **P = 0.0049. **e**, Time to 50% activation of WT and *ALPK3*^mut^ hCOs. **f**, Time to 50% relaxation of WT and *ALPK3*^mut^ hCOs. **g**, WT and *ALPK3*^mut^ hCO R-R interval scatter of hCOs to index arrhythmicity. ****P < 0.0001. **h**, Gene structure of mouse *Alpk3* with location of the W1538X variant, which corresponds to W1765X in humans. Hematoxylin and eosin-stained coronal sections of *Alpk3*^WT/WT^ and *Alpk3*^W1538X(+/+)^ hearts at 21 d and representative M-mode and mitral valve pulse wave Doppler echocardiographs of *Alpk3*^WT/WT^ and *Alpk3*^W1538X(+/+)^ mice are shown. **i**, Summary echocardiography data of *Alpk3*^WT/WT^, *Alpk3*^W1538X(+/−)^ and *Alpk3*^W1538X(+/+)^ mice displaying left ventricular mass, ejection fraction, left ventricular systolic volume, E/A wave ratio, IVRT and myocardial performance index. For the hCO experiments, *n* = 82 and 45 hCOs for WT and *ALPK3*^mut^, respectively over four independent replicates. Statistical significance was established using a two-tailed Mann–Whitney *U*-test. The error bars represent the s.e.m. For mouse echocardiography, *n* = 9, 8 and 9 mice for *Alpk3*^WT/WT^, *Alpk3*^W1538X(+/−)^ and *Alpk3*^W1538X(+/+)^, respectively. Statistical significance was established by ordinary one-way analysis of variance (ANOVA) with Tukey’s multiple-comparisons test. The error bars represent the s.e.m. Source data

**Extended Data Fig. 4:**
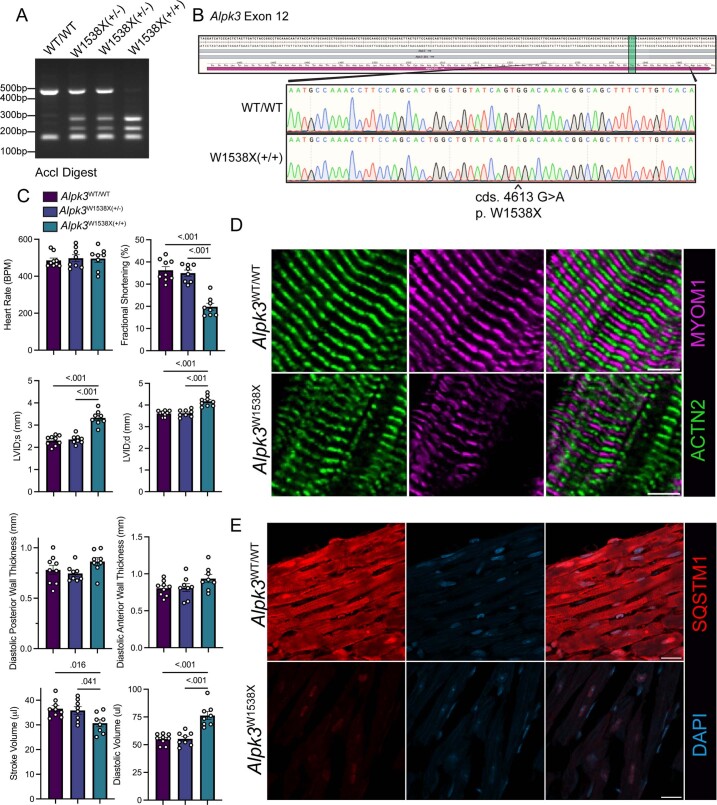
Generation and characterization of *Alpk3*^W1538X^ mouse model. **a** Representative AccI digest of genotyping PCR products from *Alpk3*^WT/WT^, *Alpk3*^W1538X(+/−)^, and *Alpk3*^W1538X(+/+)^ mice. **b** Sanger sequencing chromatograms of WT and *Alpk3*^W1538X(+/+)^ mice against reference c57/Bl6 mouse *Alpk3* gene. **c** Summary echocardiography data (continued from Fig. 3i) of 6-week-old mice showing heart rate, left ventricular diastolic and systolic internal diameter, fractional shortening, diastolic posterior and anterior wall thickness, stroke volume, and diastolic volume. Statistical significance established by ordinary one-way ANOVA with Tukey’s multiple comparison test. Error bars represent S.E.M. **d** Immunofluorescent staining of ACTN2 (green) and MYOM1 (magenta) in 21-dayold *Alpk3*^WT/WT^ and *Alpk3*^W1538X(+/+)^ mouse hearts. Scale bar = 5 µm. **e** Immunofluorescent staining of SQSTM1 (red) and DAPI (blue) in 21-day-old *Alpk3*^WT/WT^ and *Alpk3*^W1538X(+/+)^ mouse hearts. Scale bar = 15 µm. Source data

Human cardiac organoids (hCOs) were generated^28^ to assess changes in contractile function between WT and *ALPK3*^mut^ heart cells (Fig. 3a,b). Systolic force generation was significantly reduced in *ALPK3*^mut^ hCOs (Fig. 3c) together with a reduction in beating rate (Fig. 3d). Although no changes in contraction or relaxation kinetics were observed (Fig. 3e,f), *ALPK3*^mut^ hCOs were arrhythmogenic (Fig. 3g). Immunofluorescence staining of hCOs for the Z-disc marker ACTN2 and M-band marker OBSCN showed that sarcomeric organization, particularly at the M-band, was disrupted in *ALPK3*^mut^ hCOs (Extended Data Fig. 2i). In addition, as observed in two-dimensional myocytes, some aggregation of the M-band component OBSCN and the Z-disc component ACTN2 was apparent in *ALPK3*^mut^ hCOs (Extended Data Fig. 2i). To test our findings that ALPK3 regulates cardiac contractility in vivo, we generated a mouse model containing the variant W1538X (*Alpk3*^W1538X^), analogous to the patient-specific variant *ALPK3* c.5294G>A, p.W1765X, causing a truncation in the proximal region of the alpha kinase domain and occurring in a family affected by HCM (Extended Data Fig. 4a,b)^13^. At as early as 3 weeks old, mice homozygous for the variant displayed global enlargement of cardiac chambers by histology (Fig. 3h). Systolic dysfunction and cardiac enlargement were evident by echocardiography at 6 weeks of age (Fig. 3i and Extended Data Fig. 4c). Additionally, transmitral valve Doppler echocardiography showed severe diastolic dysfunction in *Alpk3*^W538X^ mice as indicated by significantly reduced E/A wave ratio and longer isovolumic relaxation times (IVRTs) (Fig. 3i and Extended Data Fig. 4c). Immunofluorescence revealed sarcomeric disarray, particularly at the M-band, as indicated by disorganization of MYOM1 (Extended Data Fig. 4d). Collectively, these results highlight the requirement of ALPK3 to maintain force generation, sarcomere integrity and beating rhythmicity in 3D human cardiac tissue.

### ALPK3 deficiency disrupts key cardiac protein networks

**Extended Data Fig. 5:**
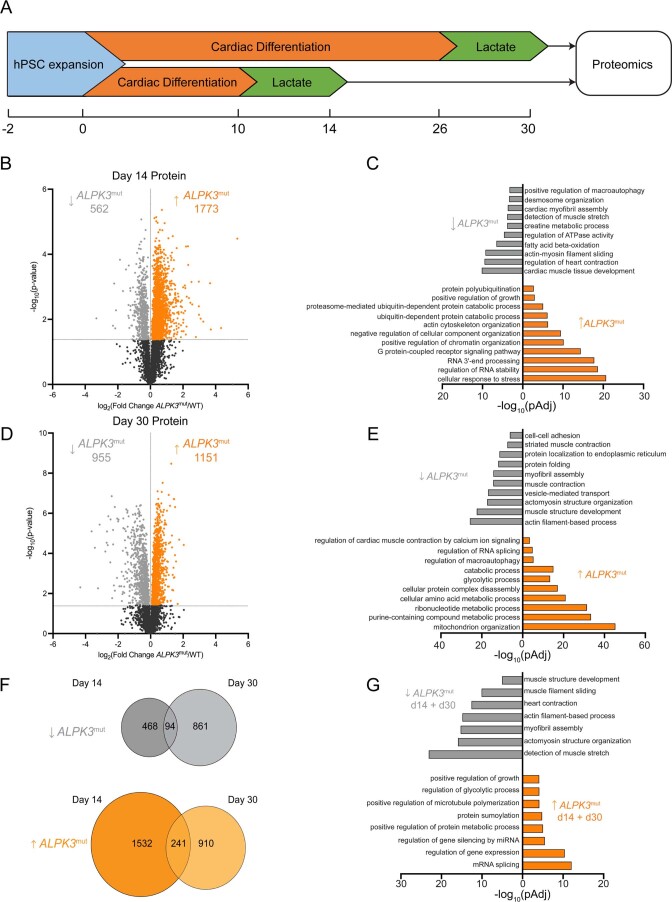
ALPK3 deficient cardiomyocytes have compromised expression of key cardiac protein networks. **a** Experimental outline of proteomics experiment. n = 5 per group. **b** Volcano plot of day 14 WT and *ALPK3*^mut^ hPSC-CMs to show differential protein expression. **c** Gene ontology (GO) of biological processes enriched in differentially expressed proteins in day 14 WT and *ALPK3*^mut^ hPSC-CMs**. d** Volcano plot of day 30 WT and *ALPK3*^mut^ hPSC-CMs to show differential protein expression. **e** GO of biological processes enriched in differentially expressed proteins in day 30 WT and *ALPK3*^mut^ hPSC-CMs. **f** Venn diagrams of protein expression up- or down-regulated proteins common for day 14 and day 30. **g** GO of biological processes of commonly up- or down-regulated proteins in *ALPK3*^mut^ hPSC-CMs.

**Extended Data Fig. 6:**
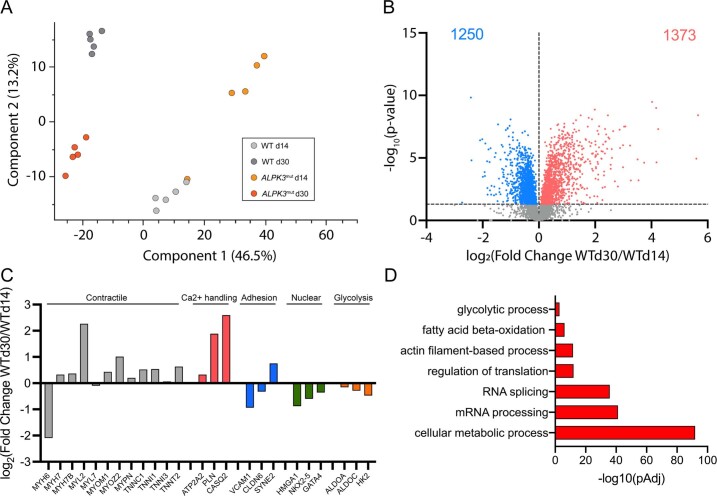
Proteomic profile of day 14 vs. day 30 WT hPSC-CMs. **a** Principal component analysis plot of day 14 and day 30 purified WT and *ALPK3*^mut^ hPSC-CMs. **b** Volcano plot of proteins demonstrating log fold change of day 30 proteins vs. day 14 proteins in WT hPSC-CMs. **c** Protein expression profile of select contractile, calcium handling, adhesion, nuclear, and glycolytic proteins at day 30 vs. day 14. **d** GO term biological processes associated with changes in protein between day 30 and day 14 WT hPSC-CMs. Source data

To define the ALPK3-dependent molecular networks, we compared the proteomic profiles of purified WT and *ALPK3*^mut^ hPSC CMs (Extended Data Fig. 5a) at days 14 (early) and 30 (late) of cardiac differentiation (Extended Data Fig. 6). Principal-component analysis demonstrated good reproducibility between replicates, with maturation-dependent changes in the proteomic signature of *ALPK3*^mut^ CMs (Extended Data Fig. 4a). We investigated altered biological processes at days 14 (2,335 differentially expressed proteins; Extended Data Fig. 5b) and 30 (2,106 differentially expressed proteins; Extended Data Fig. 5d). At both time points, pathways related to muscle structure, contraction and stretch-sensing were downregulated in *ALPK3*^mut^ CMs (Extended Data Fig. 5c,e). Furthermore, *ALPK3*^mut^ hPSC CMs exhibited deregulation of pathways related to protein quality control (autophagy, protein ubiquitination, sumoylation) and metabolism (glycolysis, fatty acid oxidation, creatine and ribonucleotide metabolism). Integration of early and late time points showed divergence of many differentially expressed proteins (Extended Data Fig. 5f), demonstrating maturation- or phenotype-dependent shifts in *ALPK3*^mut^ myocytes. Of the 335 proteins commonly upregulated or downregulated at both time points (Extended Data Fig. 5f), pathways that regulate heart development and contraction, sarcomere organization and stretch detection were reduced in *ALPK3*^mut^ CMs (Extended Data Fig. 5g), while cell growth, metabolism, gene expression and microtubule polymerization pathways were enriched (Extended Data Fig. 5g). These data collectively suggest that ALPK3 contributes to M-band signaling. Importantly, the M-band is understood to be a mechanosensitive regulator of sarcomere organization^29^, muscle metabolism^30^ and protein turnover^8^.

**Extended Data Fig. 7:**
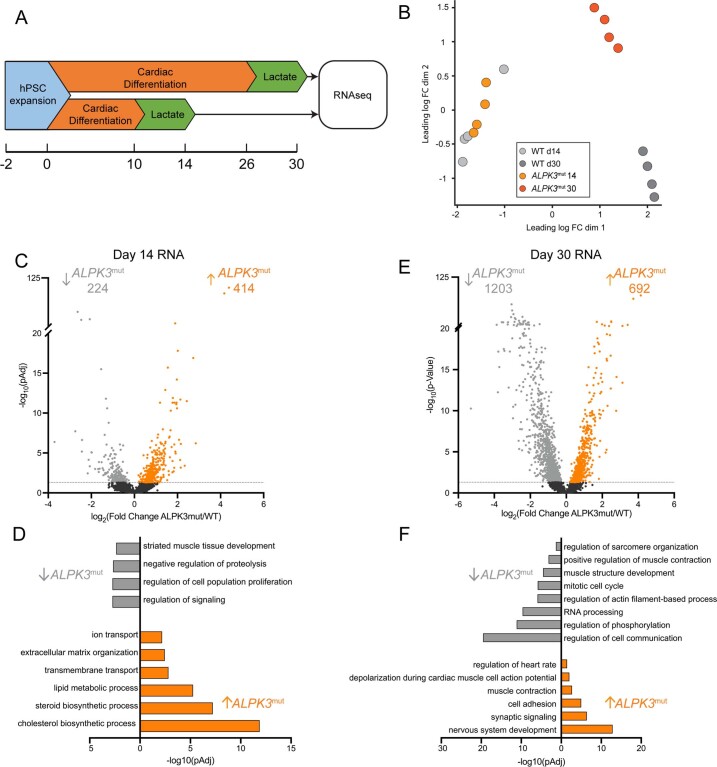
RNA sequencing of purified WT and *ALPK3*^mut^ hPSC-CMs. **a** Experimental outline. n = 4 per group. **b** Principal component analysis of day 14 and 30 WT and *ALPK3*^mut^ hPSC-CMs. **c** Volcano plot of transcript levels between day 14 *ALPK3*^mut^ and WT hPSC-CMs. **d** GO term analysis of enriched biological processes of differentially expressed transcripts between day 14 *ALPK3*^mut^ and WT hPSC-CMs. **e** Volcano plot of transcript levels between day 30 *ALPK3*^mut^ and WT hPSC-CMs. **f** GO term analysis of enriched biological processes of differentially expressed transcripts between day 30 *ALPK3*^mut^ and WT hPSC-CMs.

Further, RNA-seq analysis revealed that transcriptional differences between WT and *ALPK3*^mut^ hPSC CMs were less pronounced at day 14 than at day 30, suggesting that transcriptional remodeling is secondary to dysregulation of the ALPK3-dependent proteome (Extended Data Fig. 6a,b). RNA-seq data, at both days 14 and 30, identified a suite of commonly downregulated genes in *ALPK3*^mut^ hPSC CMs that were enriched in biological processes related to heart development, contraction and sarcomeric organization (Extended Data Fig. 6c–f). The broad reduction in contractile protein levels was evident, albeit to a lesser extent, in the RNA-seq data (Extended Data Fig. 7). These data suggest that post-transcriptional processes predominantly drive the phenotypic responses in *ALPK3* mutant myocytes.

### ALPK3 is critical for the phosphorylation of sarcomeric and protein quality control proteins

**Fig. 4:**
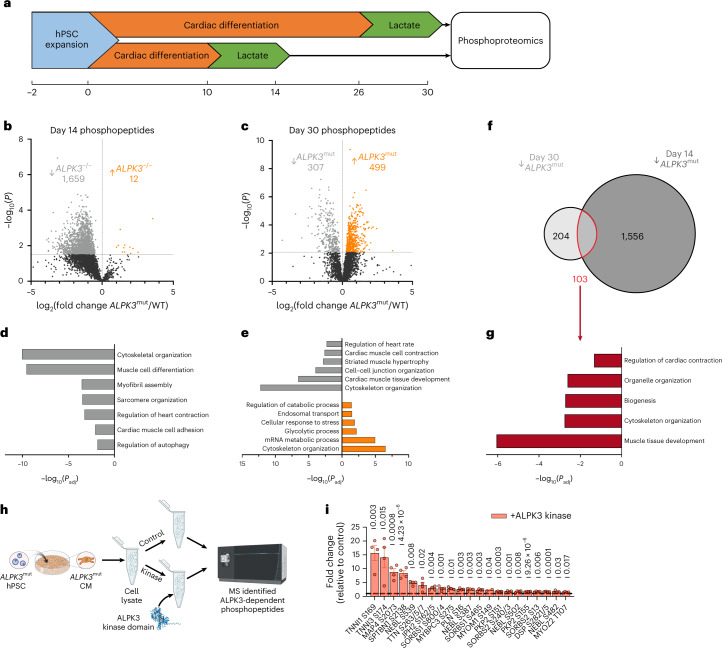
ALPK3 is critical for phosphorylation of sarcomeric and autophagy components. **a**, Experimental outline of the phosphoproteomic experiments; *n* = 5 independent differentiations per group. **b**, Volcano plot of day 14 WT and *ALPK3*^mut^ hPSC CMs to show differential abundance of normalized phosphopeptides. **c**, Volcano plot of WT and *ALPK3*^mut^ hPSC CMs to show the differential abundance of normalized phosphopeptides at day 30. **d**, GO terms of biological processes enriched in differentially phosphorylated proteins between day 14 WT and *ALPK3*^mut^ hPSC CMs. **e**, GO terms of biological processes enriched in differentially phosphorylated proteins between day 30 WT and *ALPK3*^mut^ hPSC CMs. **f**, Venn diagram showing the overlap of dephosphorylated phosphopeptides in *ALPK3*^mut^ hPSC CMs between day 14 and day 30. **g**, GO terms of biological processes enriched in commonly dephosphorylated proteins in *ALPK3*^mut^ hPSC CMs between day 14 and day 30. **h**, Schematic of ALPK3 global kinase assay performed in *ALPK3*^mut^ hPSC CMs. **i**, Cytoskeletal and contractile proteins with elevated phosphopeptide abundance after the ALPK3 kinase assay. *n* = 2–4 per condition. The dashed bar indicates that the control value = 1. Values represent unadjusted *P* values from a two-sided *t*-test. Data are shown as the mean ± s.e.m. Source data

To understand potential ALPK3-dependent signaling pathways, we compared the global phosphoproteomic profile of purified WT and *ALPK3*^mut^ CMs, again at two points of differentiation (Fig. 4a). We detected 4,211 phosphorylated peptides with 1,671 and 806 peptides, normalized to total protein abundance, differentially phosphorylated at days 14 and 30, respectively (Fig. 4b,c). At day 14, 1,659 peptides from 526 unique proteins were dephosphorylated in *ALPK3*^mut^ myocytes, which were associated with loss of pathways related to sarcomere assembly, muscle contractility and cell adhesion (Fig. 4d). Autophagy components were dysregulated, suggesting either that this may be a generalized stress response^31^ or that ALPK3 signaling may contribute to the regulation of protein quality control in CMs. Only 12 phosphopeptides from 12 unique proteins were increased in *ALPK3*^mut^ CMs at day 14 (Fig. 4b). Although the number of dephosphorylated peptides was lower at day 30 (1,659 versus 307), the set of 154 unique proteins identified was also enriched in Gene Ontology (GO) terms related to cytoskeletal organization, heart contraction and cell adhesion (Fig. 4e). At the day 30 time point (Fig. 4c), the number of enriched phosphopeptides observed in *ALPK3*^mut^ CMs was dramatically higher than at day 14 (499 versus 12; the 499 peptides represent 271 unique proteins), suggesting that increased phosphorylation is a compensatory signaling response to extended stress with enriched processes including stress response, glycolysis, RNA metabolism and endosome transport (Fig. 4e).

There were 103 peptides from 58 unique proteins that were significantly dephosphorylated in *ALPK3*^mut^ hPSC CMs at both the early and late time points (Fig. 4f ). In agreement with the *ALPK3* cardiomyopathy phenotype^13,17^ and impaired contractility (Figs. 2 and 3), commonly dephosphorylated proteins were enriched in the regulation of the cardiac contraction and cytoskeletal organization pathways (Fig. 4g). Taken together, these data suggest that ALPK3 contributes, either directly or indirectly, to the phospho-regulation of key cytoskeletal proteins to maintain sarcomere organization and function.

**Extended Data Fig. 8:**
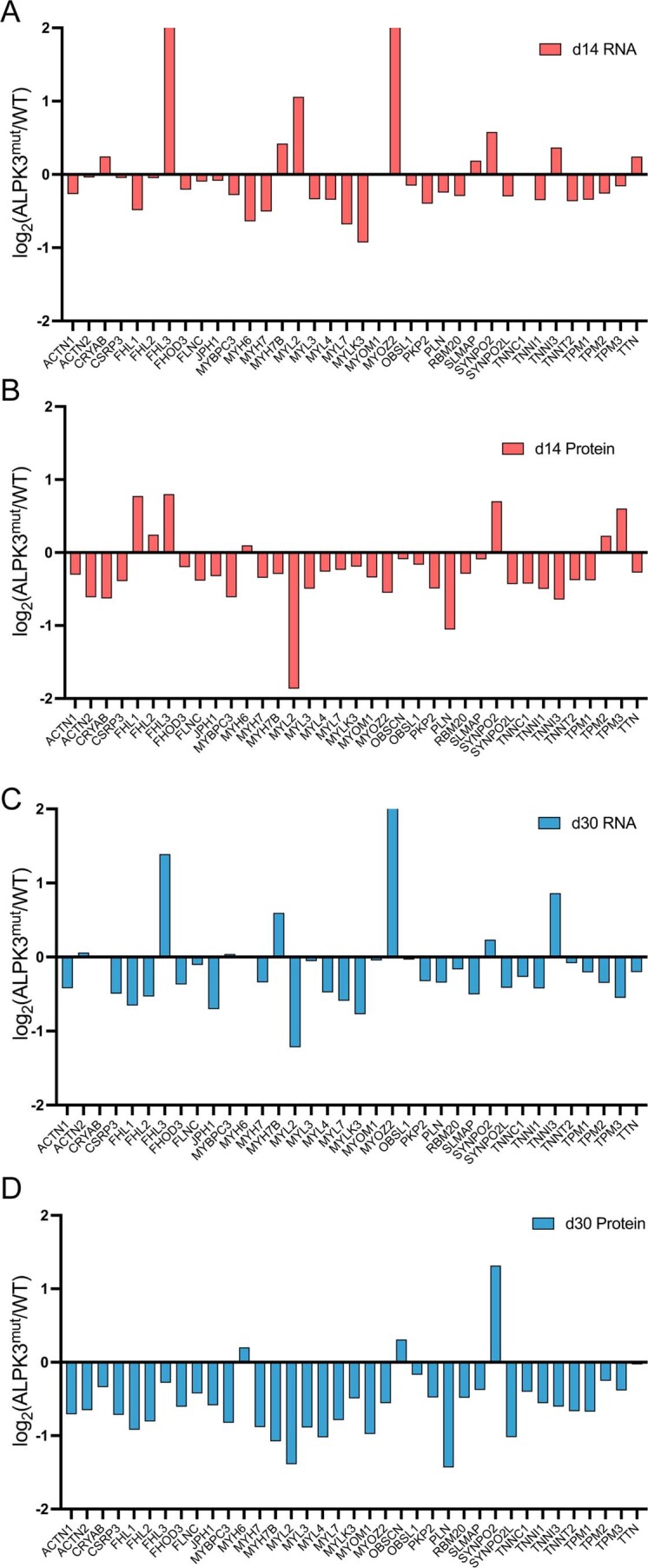
Comparison of RNA and protein expression of contractile proteins in WT and ALPK3^mut^ purified hPSC-CMs at two timepoints. **a**. Logarithmic change of RNA transcript levels between day 14 WT and *ALPK3*^mut^ hPSC-CMs. n = 4 per group. **b**. Logarithmic change of protein levels between day 14 WT and *ALPK3*^mut^ hPSCCMs. n = 5 per group. **c**. Logarithmic change of RNA transcript levels between day 30 WT and *ALPK3*^mut^ hPSC-CMs. n = 4 per group. **d**. Logarithmic change of protein levels between day 30 WT and *ALPK3*^mut^ hPSCCMs. n = 5 per group. Source data

**Extended Data Fig. 9:**
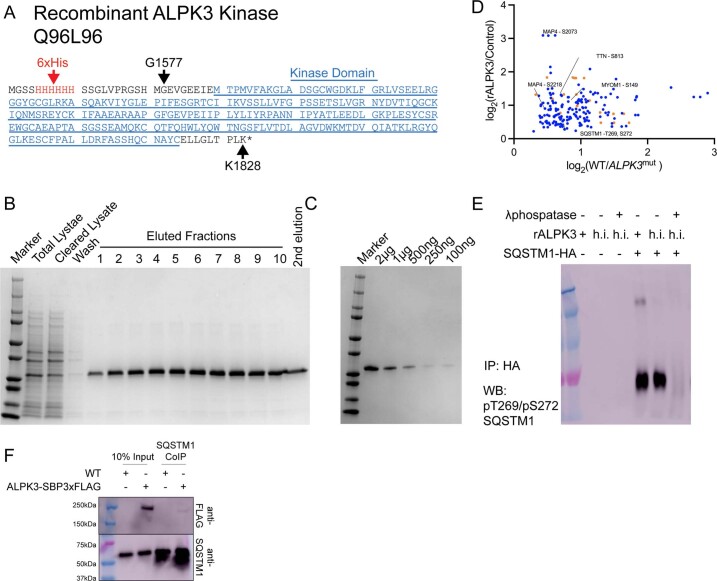
Expression and purification of recombinant ALPK3 kinase domain for kinase assays. **a** Sequence of recombinant ALPK3 kinase expression used for Pet28a + *e. coli* expression. **b** Nickel agarose affinity purification of 6xHis-tagged recombinant ALPK3 kinase. **c** Representative gel of recombinant ALPK3 kinase following dialysis and concentration. **d** Scatter plot of peptides phosphorylation in *ALPK3*^mut^ by recombinant kinase ALPK3 (fold-change >0, p < 0.1) versus peptides significantly dephosphorylated in day 14 *ALPK3*^mut^ hPSC-CMs compared to WT (fold-change >0, q < 0.05). Orange points represent proteins associated with cardiomyopathy by KEGG. **e** *In vitro* phosphorylation of SQSTM1 by recombinant ALPK3 kinase. **f** ALPK3-SBP-3XFLAG co-immunoprecipitation of endogenous SQSTM1 followed by Western blot for FLAG or SQSTM1. Source data

To further probe the ALPK3-dependent phosphoproteome, we isolated the recombinant ALPK3 kinase domain for biochemical studies (Extended Data Fig. 9a–c). A global kinase assay was performed in phosphorylation-depleted, day 14 *ALPK3*^mut^ hPSC CMs (Fig. 4h). Before the assay, lysates were treated with an irreversible pan-kinase inhibitor to block endogenous kinase activity^32,33^. When recombinant ALPK3 kinase was applied to lysates, we identified 500 phosphosites that were significantly elevated compared to controls. Notably, a number of cytoskeletal, contractile or proteostasis proteins exhibited greater phosphorylation when lysates were treated with ALPK3 (Fig. 4i and Extended Data Fig. 9d). Correlation between this global kinase assay and day 14 phosphoproteomics data demonstrated agreement between proteins dephosphorylated in *ALPK3*^mut^ CMs and proteins that could be phosphorylated by recombinant ALPK3 (Extended Data Fig. 8d). These findings favor the hypothesis that ALPK3 is a myogenic kinase that signals via the sarcomeric M-band.

### ALPK3 is required for the sarcomeric localization of SQSTM1

**Fig. 5:**
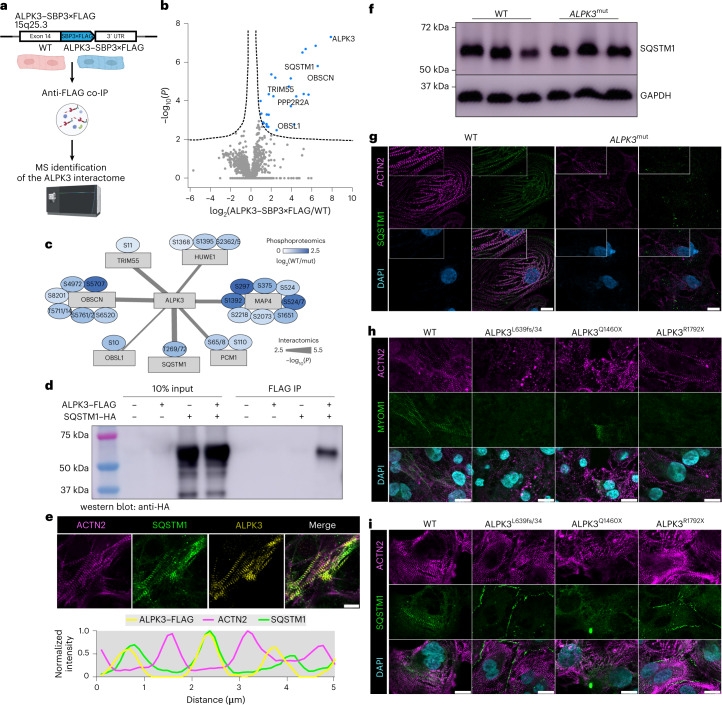
ALPK3 binds the autophagy regulatory SQSTM1 (p62) and is required for the sarcomeric localization of SQSTM1. **a**, Outline of the co-IP experiment to identify ALPK3 interactors. Created with BioRender.com. **b**, Volcano plot of enriched peptides in ALPK3–SBP3×FLAG hPSC CMs identified by mass spectrometry; *n* = 5 per group. **c**, Network integration of ALPK3-bound proteins containing significantly dephosphorylated phosphosites in *ALPK3*^mut^ hPSC CMs. **d**, HEK 293FT co-IP of ALPK3–FLAG and SQSTM1–HA. **e**, Immunofluorescence staining of ALPK3–FLAG (yellow), SQSTM1 (green) and α-actinin (magenta) in ALPK3–SBP3×FLAG hPSC CMs and intensity plot of all three stains. Scale bar, 15 μm. **f**, Western blot of SQSTM1 levels in WT and *ALPK3*^mut^ hPSC CMs. **g**, Representative immunofluorescence staining of α-actinin (magenta), SQTSM1 (green) and DAPI (blue) in WT and *ALPK3*^mut^ hPSC CMs. Scale bars, 15 μm. **h**, Immunofluorescence staining of MYOM1 (green), α-actinin (magenta) and DAPI (blue) in WT and *ALPK3* patient-specific variant hPSC CMs. Scale bars, 15 μm. **i**, Immunofluorescence localization of SQSTM1 (green), α-actinin (magenta) and DAPI (blue) in WT and *ALPK3* patient-specific variant hPSC CMs. Scale bars, 15 μm. Source data

**Extended Data Fig. 10:**
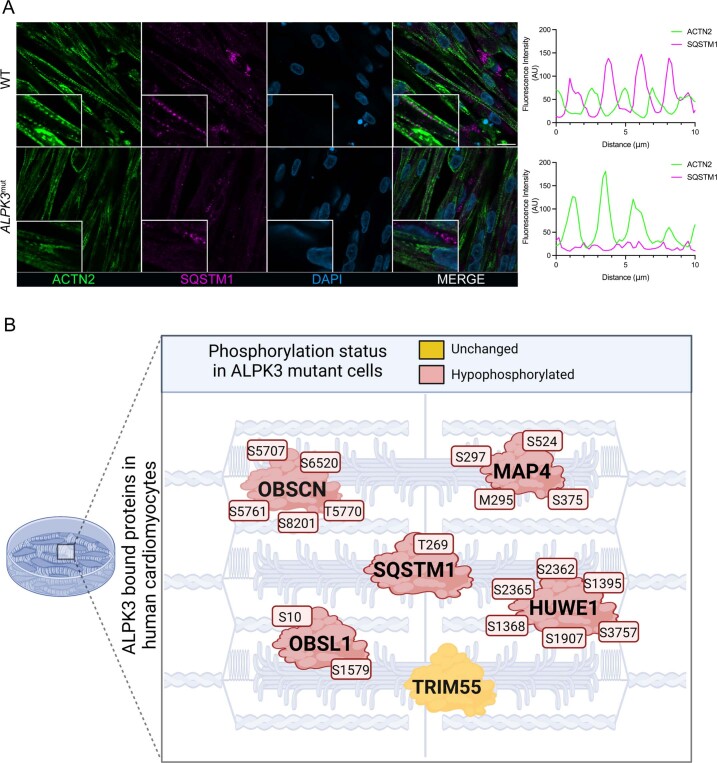
SQSTM1 localization in WT and *ALPK3*^mut^ hPSC-derived skeletal muscle cells. **a**. Immunofluorescent micrograph of WT and *ALPK3*^mut^ hPSC-skeletal muscle stained for alpha actinin (green), SQSTM1 (magenta), and DAPI (blue). Scale bar = 15 µm. Line profile demonstrating alpha actinin and SQSTM1 organization. **b**. Graphical representation of select proteins bound to ALPK3 which also showed reduced phosphopeptide abundance in *ALPK3*^mut^ hPSC-CMs. The affected phosphorylation sites are also highlighted. Created with BioRender.com.

Our phosphoproteomic analyses indicated that ALPK3 deficiency caused dephosphorylation of many proteins associated with sarcomere organization and function as well as protein quality control. To identify the ALPK3 protein network, we performed mass spectrometry on proteins enriched by co-IP with endogenous ALPK3 carrying a three-tandem-repeat FLAG tag (Fig. 5a and Extended Data Fig. 2e). Together with ALPK3, 25 proteins were enriched with endogenously FLAG-tagged ALPK3 hPSC CMs over controls (Fig. 5b). Consistent with ALPK3’s intracellular localization (Fig. 1d,e), several known M-band proteins associated with ALPK3, such as OBSCN and obscurin-like protein 1 (OBSL1), demonstrating the fidelity of this co-IP experiment (Fig. 1b,c). In addition to the sarcomeric proteins, ALPK3 interacted with proteostasis proteins such as the E3 ligase MuRF2 (TRIM55) and the ubiquitin-binding protein SQSTM1 (p62). Importantly, several ALPK3-bound proteins also demonstrated reduced phosphopeptide abundance (Fig. 5c and Extended Data Fig. 10b) and have been associated with muscle pathology including OBSCN^34^, OBSL1 (ref. ^35^), SQSTM1 (ref. ^36^) and HUWE1 (ref. ^37^). As both MuRF2 and SQSTM1 are known to interact with titin kinase at the M-band to regulate mechanosensitive signaling and protein turnover^8^, we further investigated the ALPK3–SQSTM1 interaction. We first validated our finding by performing reverse co-IP and found that ALPK3 immunoprecipitated SQSTM1 in ALPK3–SBP3×FLAG hPSC CMs (Extended Data Fig. 9f). We then tested the interaction using a heterologous, nonmuscle system that confirmed the association between ALPK3 and SQSTM1 when these proteins were overexpressed in HEK 293 cells (Fig. 5d). Furthermore, ALPK3 and SQSTM1 colocalized at the M-band of hPSC CMs (Fig. 5e). While total SQSTM1 levels were unchanged between WT and *ALPK3*^mut^ cultures (Fig. 5f), ALPK3 demonstrated the capacity to phosphorylate SQSTM1 (Extended Data Fig. 9d,e) and SQSTM1 was dislocated from the sarcomere and was localized to cytosolic aggregates in *ALPK3*^mut^ hPSC-derived cardiac and skeletal muscle cells (Fig. 5g and Extended Data Fig. 10). To determine whether M-band organization and SQSTM1 localization may underlie pathogenesis in *ALPK3*-associated HCM, we generated three additional hPSC lines harboring *ALPK3* variants (L639fs/34, Q1460X, R1792X) from a recently published cohort of patients with HCM^16^. On differentiation into CMs, cells expressing each of these *ALPK3* patient-specific variants recapitulated the key pathological features of sarcomere disorganization and loss of M-band MYOM1 (Fig. 5h). Furthermore, SQSTM1 was not detected in the sarcomeres of these patient-specific variant hPSC lines but formed aggregates either within the cytosol or at the cell membrane (Fig. 5i). Finally, the sarcomeric localization of SQSTM1 was compromised in the myocardium from the *Alpk3*^W1538X^ mouse model (Extended Data Fig. 4e). Collectively, these data suggest that the binding of ALPK3 to SQSTM1 is required for M-band localization of SQSTM1 in striated muscle and disruption of the intracellular localization of SQSTM1 may be a prominent mechanism driving ALPK3-related HCM. Thus, ALPK3 is integral to M-band integrity and signaling as illustrated by the disorganization of MYOM1 (Figs. 2b and 5h and Extended Data Fig 4), OBSCN (Extended Data Fig. 2i) and SQSTM1 (Fig. 5g,h and Extended Data Fig. 4e) in ALPK3-deficient myocytes.

**Fig. 6:**
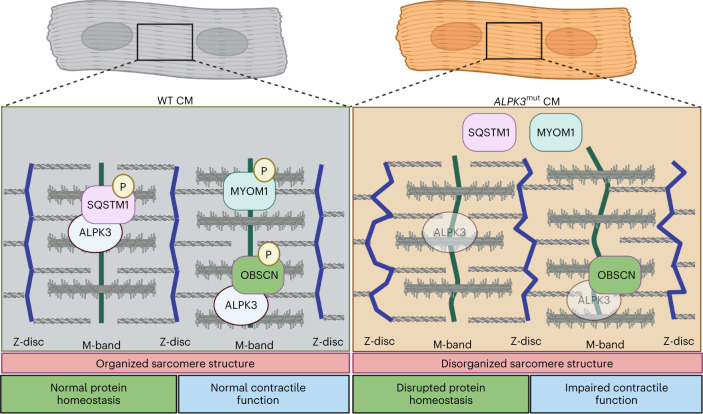
Graphical representation of the findings from this study. Graphical summary of key findings from this study of sarcomere disorganization and SQSTM1 and MYOM1 mislocalization in *ALPK3*^mut^ hPSC CMs. Created with BioRender.com.

## Discussion

Our data show that ALPK3 is a myogenic kinase that localizes to the M-band of sarcomeres in striated muscle. We define the ALPK3-dependent phosphoproteome in hPSC-derived CMs at two stages of differentiation. Among the proteins that require ALPK3 for phosphorylation are components of the sarcomere, the functional unit that generates the force underpinning muscle contraction. In addition, the phosphorylation status of the cellular protein quality control system is also disrupted in ALPK3-mutant CMs. In this context, we identify that SQSTM1 and MuRF2 both interact with ALPK3 and may provide a mechanism whereby the M-band has a key role in detecting and removing damaged proteins from the sarcomere. Furthermore, ALPK3 is necessary for the M-band localization of SQSTM1. In conclusion, these findings support a major role for ALPK3 in striated muscle contraction and the intracellular signaling networks regulating CM contractility.

Altered CM mechanotransduction is a common feature of cardiomyopathy^38^. While this has been investigated extensively in the context of the Z-disc signaling pathways^6,39–41^, comparatively little is understood about the contribution of M-band biology. Our findings suggest that ALPK3 is a component of the sarcomeric M-band (Fig. 6). A recent study supports this localization^21^ but also reported a nuclear localization that we did not observe. This nuclear localization may be highly context-dependent. Notably, previously published localization studies relied on transient overexpression of the protein in nonmuscle cells or hPSC-derived CMs rather than endogenous tagging of the protein used in this study^20,21^. Further, ALPK3 is critical for M-band integrity as the established M-band marker MYOM1 was not detected at the sarcomere of ALPK3-mutant CMs. This loss of M-band MYOM1 was also observed in CMs harboring HCM-associated *ALPK3* variants and was highly disorganized in a mouse model of ALPK3 cardiomyopathy. We propose that ALPK3 forms a signaling node at the M-band, which is required to maintain sarcomere integrity. Dysregulation of M-band proteins, including ultrastructural deficits in M-band and thick filament organization, were also observed in a recent independent study^21^. The titin kinase signalosome is the best understood pathway at the M-band. In this pathway, mechanical stretch induces a conformational change in the kinase domain, which recruits the quality control proteins NBR1, SQSTM1 and MuRF2 to the M-band^8,42,43^. This pathway regulates cardiac proteostasis in response to mechanical stimuli via MuRF2 regulation of *SRF* gene expression and regulation of SQSTM1 localization^7^. Modeling of ALPK3, based on the crystal structure of another alpha kinase TRPM7, has posited that ALPK3 lacks catalytic activity; thus, it was hypothesized to act as a pseudokinase^21^. In contrast, by means of an unbiased global kinase assay, we found that ALPK3 kinase treatment increased phosphorylation of many proteins, including SQSTM1, which we validated as a direct target. Thus, our study supports the hypothesis that ALPK3 possesses catalytic activity. Future studies including a detailed crystal structure of ALPK3 to define the residues that may regulate catalytic activity are critical. Our data indicate that ALPK3 may form a signaling network with titin kinase, which also binds SQSTM1 and MuRF2 at the M-band, to link mechanical signals to protein quality control networks. For example, the ALPK3–SQSTM1 interaction is required to maintain the sarcomeric localization of SQSTM1, with ALPK3 deficiency leading to mislocalization of SQSTM1 and impaired contractility in hPSC-derived CMs. Additionally, ALPK3 can phosphorylate SQSTM1; this supports the hypothesis that this is mediated through a direct interaction. However, it is possible that this is mediated indirectly. Future studies are required to address the nature of this interaction and its physiological consequences.

Given the longevity of human cardiac muscle cells, efficient protein quality control mechanisms are essential to maintain CM function^44^. The hypertrophic heart experiences sustained biomechanical and oxidative stress. In the myocyte, this translates to increased strain on contractile sarcomere proteins and higher rates of protein misfolding^45^. While this misfolding is initially compensated, protein quality control mechanisms cannot maintain the sustained activity required for normal heart function. Indeed, aberrant protein quality control is a common feature of HCM^31,45–47^, which eventually leads to compromised cardiac structure and function. In this context, the observation that ALPK3 interacts with regulators of protein homeostasis such as MuRF2 and SQSTM1 within the M-band suggests a role in controlling sarcomeric proteostasis. This model of ALPK3 activity is analogous to that seen for titin kinase at the M-band^7,12^ and BAG3 at the Z-disc^6^, suggesting that sarcomeric integrity is underpinned by the complex interplay between several regulatory pathways. Given our results, which demonstrate mislocalization of SQSTM1 in three independent ALPK3 pathogenic variants, and the growing number of ALPK3 variants linked to HCM^15,16^, our findings point to a central role for disrupted sarcomeric homeostasis in cardiomyopathy. Although our findings provide support for a biochemical link between ALPK3 and established regulators of proteostasis (SQSTM1, TRIM55), further studies are required to directly establish whether sarcomeric protein turnover is disrupted due to *ALPK3* mutations.

Our study demonstrates that ALPK3 is required to maintain sarcomere integrity and contractile function in both primary mouse and hPSC-derived CMs. Our findings define ALPK3 as a key functional component of the M-band in striated muscle. In addition, ALPK3 has a role in regulating protein quality control pathways via interactions with SQSTM1 and MuRF2 at the M-band. Given the dysregulation of protein quality control networks in cardiac disease, ALPK3 may be a promising therapeutic target to restore heart function in cardiomyopathies.

## Methods

### Human ethics statement

All experiments were approved by the Royal Children’s Hospital Research Ethics Committee (HREC 33001A). Methods were carried out in accordance with the relevant guidelines and regulations provided by National Health and Medical Research Council (National Statement on Ethical Conduct in Human Research).

### Statistics and reproducibility

The investigators were blinded to genotype during the experiments and analysis. No statistical method was used to predetermine sample size. No data were excluded from the analyses. The experiments were not randomized. For confocal microscopy analysis, representative images are shown and experiments were performed a minimum of two times with the experimenter blinded to genotype.

### snRNA-seq bioinformatics analysis

snRNA-seq data analysis has been published previously^25,48^. The bioinformatics analysis pipeline is available at http://www.heartexplorer.org/ including links retrieved from the analysis website and the source code. Briefly, DoubletFinder (v.2.0.3)^49^ was used to remove doublets. Subsequently, gene filtering discarded mitochondrial and ribosomal genes, as well as genes that were not annotated. Genes that had at least one count in at least 20 nuclei were retained for further analysis, assuming a minimum cluster size of 20 nuclei. All genes on the X and Y chromosomes were removed before clustering and all subsequent analyses. We performed SCTransform normalization^50^, data integration^50–52^, data scaling and graph-based clustering separately using the R package Seurat (v.3.0.2). Data integration of the biological replicates for each group was performed using CCA^3^ from the Seurat package with 30 dimensions and 3,000 integration anchors followed by data scaling. Clustering of the nuclei was performed with 20 principal components and an initial resolution of 0.3. Marker genes to annotate clusters were identified as significantly upregulated genes for each cluster using moderated *t*-tests, accounting for the mean variance trend and employing robust empirical Bayesian shrinkage of the variances, followed by TREAT tests specifying a log fold change threshold of 0.5 and false discovery rate (FDR) cutoff of <0.05, using the R package limma (v.3.40.2).

### Stem cell culture and cardiac differentiation

The female HES3 (ref. ^53^) (WiCell ES03) *NKX2-5*^eGFP/+^ human embryonic stem cell (hESC) line was used for all experiments and has been described previously^54^. The *ALPK3*^mut^ loss-of-function hPSC line was generated previously using CRISPR–Cas9 (ref. ^17^). Stem cells were routinely passaged using TrypLE (cat. no. 12604013, Thermo Fisher Scientific) onto Geltrex-coated flasks (cat. no. A1413301, Thermo Fisher Scientific) and mTeSR plus medium (cat. no. 05825, STEMCELL Technologies). The selective ROCK1 inhibitor Y-27632 (cat. no. S6390, Selleck Chemicals) was used when passaging. Differentiation into hPSC CMs was performed using a monolayer culture system with a small-molecule Wnt activation/inhibition protocol^25^. Briefly, stem cells were plated on day −2 at 20,000 cells per cm^2^ with mTeSR plus (with 10 mM of Y-27632). The next day, the medium was refreshed (without Y-27632). On day 0 of the differentiation, the medium was switched to basal differentiation medium (RPMI 1640 supplemented with 2% B-27 minus vitamin A, 1% GlutaMAX, 1% penicillin-streptomycin) plus 80 ng ml^−1^ Activin A (cat. no. 338-AC, R&D Systems), 8 mM CHIR 99021 (cat. no. 4423, Tocris) and 50 μg ml^−1^ ascorbic acid (cat. no. A5960, Sigma-Aldrich). Twenty-four hours later, the medium was replaced with fresh basal differentiation medium. On day 3, the medium was exchanged to basal differentiation medium containing 5 mM of IWR-1 (cat. no. I0161, Sigma-Aldrich) and 50 μg ml^−1^ of ascorbic acid. Seventy-two hours later, cells were switched back to basal differentiation medium and maintained with medium changes every 48 h. To enrich for myocyte populations, cardiac differentiation was treated for 96 h with glucose-free DMEM containing 5 mM sodium l-lactate, 1% GlutaMAX and 1% penicillin-streptomycin, with medium exchange at 48 h.

### Genome editing

Genome editing was performed using CRISPR–Cas9. To generate 3′-tagged ALPK3 cell lines, the guide sequence was cloned into the vector pSpCas9(BB)-2A-eGFP. (The PX458 plasmid was a gift from F. Zhang, cat. no. 48138, Addgene.) Homology-directed repair templates were designed to contain 1,000-bp homology arms flanking the region to be edited. HES3 *NKX2-5*^eGFP/+^ hESCs were nucleofected with the PX458 and repair plasmids. Annealed oligonucleotides were also cloned into the pSpCas9(BB)-2A-eGFP vector for guide RNA sequences in the *ALPK3* patient-specific variant cell lines. Homology-directed repair templates were approximately 80-mer single-stranded oligodeoxynucleotides containing variants plus synonymous variants to prevent recutting by Cas9 of correctly targeted DNA. Details of the sequences used for cell line generation are in the Supplementary Information. HES3 *NKX2-5*^eGFP/+^ hESCs were cotransfected with single-stranded oligodeoxynucleotides and PX458 using Lipofectamine 3000 with the PX458 plasmid. Single green fluorescent protein (GFP)-expressing cells were sorted into 96-well plates and screened by PCR.

### Generation of the *Alpk3*^W1538X^ mouse model

The *Alpk3*^W1538X^ mouse model was generated by the MAGEC laboratory (Walter and Eliza Hall Institute) on a C57BL/6J background using CRISPR–Cas9-mediated homology-directed repair. Cas9 mRNA, single-guide RNA and repair template were injected into the cytoplasm of fertilized one-cell-stage embryos generated from WT C57BL/6J breeders. Twenty-four hours later, two-cell-stage embryos were transferred into the uteri of pseudo-pregnant female mice. Viable offspring were genotyped using next-generation sequencing. Details of the sequences used to generate the mouse model are in the Supplementary Information. Mice were fed standard chow and given water ad libitum and were maintained in a 12-h light–12-h dark cycle at ambient room temperature. All experiments were approved by the Animal Ethics Committee of the Murdoch Children’s Research Institute (A949).

### Mouse echocardiography

Echocardiography measurements were made on 6- to 8-week-old mice (*n* = 9 (3 males, 6 females), 8 (4 males, 4 females) and 9 (5 males, 4 females) for *Alpk3*^WT/WT^, *Alpk3*^W1538X(+/−)^ and *Alpk3*^W1538X(+/+)^, respectively) using a VisualSonics Vevo 3100 ultrasound machine with an MS400 18- to 38-MHz transducer (FujiFilm) under 1–2% isoflurane anesthesia. Cardiac function was reported based on parasternal short-axis M-mode imaging. Diastolic dysfunction was assessed by transmitral pulse wave Doppler flow and tissue annulus Doppler in an apical four-chamber view. All data collection and analysis were performed blinded to genotype.

### Mouse histology

Three-week-old mice were anesthetized with 4% isoflurane followed by cervical dislocation. First, mice were perfused transcardially with PBS, which was followed by 4% paraformaldehyde (PFA). Hearts were excised and drop-fixed overnight in 4% PFA. Hearts were sucrose-protected in 15% (v/v) followed by 20% (v/v) sucrose. A scalpel was used to cut hearts into two along the coronal plane and embedded in optimal cutting temperature compound. Then, 10-μm cryosections were cut and stored at −80 °C until staining.

### Flow cytometry

Cardiac differentiation was dissociated using 0.25% trypsin EDTA (cat. no. 25200056, Gibco) and filtered into a single-cell suspension. For intracellular flow, cells were fixed in 2% PFA for 10 min before permeabilization with 0.25% Triton X-100. Primary antibody staining used α-actinin (1:100 dilution, cat. nos. A7811 (Sigma-Aldrich) and ab68167 (Abcam)) or cardiac troponin T (1:100 dilution, cat. no. ab8295, Abcam) as CM markers and CD90 (1:100 dilution, cat. no. 328124, BioLegend) as a stromal cell marker. Data were acquired on a BD LSRFortessa X-20 Cell Analyzer and a BD FACSDiva (v.9.0.1) and analyzed using FlowJo (v.10.8.1) software.

### Immunofluorescence

Cells were washed with PBS before being fixed with 2% PFA for 30 min at room temperature. Before staining, cells were permeabilized in 0.1% Triton X-100 in PBS for 30 min and blocked in 5% BSA in PBS-Tween 20 (PBS-T) for 1 h. Primary antibodies were incubated overnight at 4 °C. Cells were washed three times for 5 min in PBS-T before incubation with secondary antibodies for 1 h at room temperature. For mouse hearts, cryosections were brought to room temperature and slides underwent antigen retrieval before using the same staining protocol. The complete list of antibodies used for immunofluorescence and dilutions is included in the Reporting summary. Images were analyzed with the Zen Black 2012 (v.2.3 SP1, ZEISS) and ImageJ (v.2.3.0/1.53q, NIH) software.

### Sample preparation for global (phospho)proteomics

Samples were washed three times with ice-cold PBS. Cells were then scraped off the dish using 4% (w/v) sodium deoxycholate in 100 mM Tris-HCl, pH 8.5, before heating at 95 °C for 5 min. Cell lysates were allowed to cool on ice for 5 min before snap-freezing on dry ice and were stored at −80 °C. Samples were thawed on ice, quantified with a bicinchoninic acid (BCA) assay (Thermo Fisher Scientific) and normalized to 300 µg per 200 µl. Protein was reduced to a final concentration of 10 mM Tris(2-carboxyethyl)phosphine hydrochloride (Sigma-Aldrich) and alkylated with 40 mM of 2-chloroacetamide (Sigma-Aldrich) for 5 min at 45 °C. Samples were cooled on ice and then digested with 3 µg of sequencing-grade trypsin (Sigma-Aldrich) and 3 µg of sequencing-grade Lys-C (Wako) overnight at 37 °C. A 5-µg aliquot was removed for total proteomic analysis and the phosphopeptides were enriched from the remaining digest using the EasyPhos protocol as described previously^55^.

### Sample preparation for the ALPK3 interactome

Protein G Dynabeads were resuspended in 50 µl of 2 M urea, 50 mM Tris, pH 7.5, containing 1 mM of Tris(2-carboxyethyl)phosphine hydrochloride, 5 mM of 2-chloroacetamide, 0.2 µg of trypsin and 0.2 µg of Lys-C and digested overnight at 37 °C with shaking at 1,800 r.p.m. Peptides were removed, diluted with 150 µl of 1% trifluoroacetic acid (TFA) and desalted on poly(styrenedivinylbenzene) reversed phase support micro-columns (Sigma-Aldrich) as described previously^55^. The columns were washed with 99% isopropanol containing 1% TFA followed by 5% acetonitrile containing 0.2% TFA and then eluted with 80% acetonitrile containing 1% ammonium hydroxide and dried by vacuum centrifugation.

### Expression and purification of ALPK3 recombinant kinase

The recombinant kinase domain of ALPK3 was bacterially expressed in One Shot BL21(DE3) *Escherichia coli* (Thermo Fisher Scientific). The DNA sequence encoding amino acids 1577–1828 (IDT) was subcloned into the PET-28a^+^ T7 expression vector sequenced to confirm correct insertion. For large-scale expression, 25 ml of overnight culture was shaken with lysogeny broth containing 50 μg ml^−1^ kanamycin at 37 °C until an OD_600_ reached approximately 0.8. The culture was then induced with 1 mM of isopropyl-beta-d-thiogalactopyranoside and shaken for approximately 24 h at 20 °C. Pellets were snap-frozen and stored at −80 °C. For purification, cell pellets were thawed and resuspended in *E. coli* lysis buffer (300 mM NaCl, 50 mM NaH_2_PO_4_, 10 mM imidazole, pH 8.0) with protease inhibitors and sonicated four times for 30 s at 50% with 2-min rests on ice between pulses. Lysates were cleared using centrifugation (3,200 r.p.m. for 15 min at 4 °C) and the clarified lysate was incubated with HisPur Ni-NTA resin (Pierce) to bind histidine-tagged proteins. The resin was then successively washed with wash buffer I (300 mM NaCl, 50 mM NaH_2_PO_4_, 20 mM imidazole, pH 8.0) and II (300 mM NaCl, 50 mM NaH_2_PO_4_, 50 mM imidazole, pH 8.0). Ten 1-ml fractions were collected using gravity-based column chromatography using elution buffer (300 mM NaCl, 50 mM NaH_2_PO_4_, 250 mM imidazole, pH 8.0), which was followed by a second 10-ml bulk. Pure fractions were combined for concentration and buffer exchange using 10 MWCO Protein Concentrators (Pierce).

### ALPK3 global kinase assay

Metabolically purified *ALPK3*^mut^ hPSC CMs were used to identify substrates for ALPK3; 10-cm^2^ dishes of *ALPK3*^mut^ hPSC CMs were collected in 1 ml of co-IP buffer with cOmplete, EDTA-free protease inhibitor cocktail (Roche) and PhosSTOP phosphatase inhibitor (Roche) and snap-frozen on dry ice. On the day of the assay, lysates were thawed on ice and sheared with ten strokes through a 22-gauge needle followed by three strokes through a 27-gauge needle. To irreversibly inhibit endogenous kinase activity, lysates were treated with 10 mM 5′-(4-fluorosulfonylbenzoyl)adenosine hydrochloride for 30 min at 30 °C with shaking at 1,200 r.p.m. (refs. ^32,33^). Samples were concentrated and dialyzed extensively to remove 5′-(4-fluorosulfonylbenzoyl)adenosine hydrochloride using 10 MWCO Protein Concentrators. A BCA assay was run to determine protein concentration. Each sample was split into two 0.75-mg aliquots and the volume was brought to 400 μl with co-IP buffer. An equal volume of 2× kinase assay buffer (50 mM Tris-HCl, pH 7.4, 10 mM MgCl_2_, 3 mM MnCl_2_, 2 mM dithiothreitol, 0.5 mM ATP) was added to each tube. One tube per sample received either 25 μg of recombinant ALPK3 kinase or an equivalent volume of water. Tubes were incubated at 30 °C for 3 h with shaking at 1,200 r.p.m. All samples were then snap-frozen on dry ice and stored at −80 °C until processing for mass spectrometry (MS)-based identification of phosphopeptides.

### Liquid chromatography–tandem MS acquisition

Peptides were resuspended in 2% acetonitrile containing 0.1% TFA and analyzed on a Dionex 3500 nano-HPLC coupled to an Orbitrap Eclipse mass spectrometer (Thermo Fisher Scientific) via electrospray ionization in positive mode with 1.9 kV at 275 °C and radio frequency set to 40%. Separation was achieved on a 50 cm × 75 µm column packed with C18AQ (1.9 µm, Dr. Maisch; PepSep) over 60 min at a flow rate of 300 nl min^−1^. The peptides were eluted over a linear gradient of 3–40% Buffer B (Buffer A: 0.1% formic acid; Buffer B: 80% v/v acetonitrile, 0.1% v/v formic acid) and the column was maintained at 50 °C. The instrument was operated in data-independent acquisition mode with an MS^1^ spectrum acquired over the mass range of 350–950 *m/z* (60,000 resolution, 2.5 × 10^6^ automatic gain control and 50-ms maximum injection time) followed by tandem MS analysis with a higher-energy C-trap dissociation of 37 × 16 *m/z* with 1-*m/z* overlap (28% normalized collision energy, 30,000 resolution, 1 × 10^6^ automatic gain control, automatic injection time).

### Liquid chromatography–tandem MS data processing

Data were searched against the UniProt human database (June 2021; UP000000589_109090 and UP000000589_109090_additional) with Spectronaut 15.1.210713.50606 using default parameters with peptide spectral matches, peptide and protein FDR set to 1%. All data were searched with oxidation of methionine set as the variable modification and carbamidomethylation set as the fixed modification. For the analysis of phosphopeptides, phosphorylation of serine, threonine and tyrosine was set as a variable modification. Quantification was performed using MS^2^-based extracted ion chromatograms using 3–6 fragment ions >450 *m/z* with automated fragment ion interference removal as described previously^56^.

### Proteomic and phosphoproteomic statistical and downstream analysis

Data were processed with Perseus^57^ to remove decoy data, potential contaminants and proteins only identified with a single peptide containing oxidized methionine. The ‘Expand Site’ function was additionally used for phosphoproteomic data to account for multiphosphorylated peptides before statistical analysis. For the phosphoproteomic and proteomic analysis, data were log_2_-transformed and normalized by subtracting the median of each sample. Data were filtered to contain phosphosites quantified in at least three biological replicates and statistical analysis was performed with ANOVA, including correction for multiple-hypothesis testing using a Benjamini–Hochberg FDR with *q* < 0.05 defined as a significance cutoff. For the interactome analysis, data were log_2_-transformed and normalized by subtracting the median of each sample. Data were filtered to contain proteins quantified in at least three biological replicates of the ALPK3 pull-down group. Analyses with missing data in all the replicates of the negative control group were imputed using random values from a downshifted normalized distribution of the entire dataset. Differentially enriched proteins were calculated using *t*-tests including correction for multiple-hypothesis testing using a Benjamini–Hochberg FDR with *q* < 0.05 defined as a significance cutoff.

### RNA isolation

Samples were collected in TRIzol and frozen at −80 °C until processing. RNA extraction was performed first by phase separation with chloroform (200 µl per 1 ml of TRIzol) and purified using a column-based procedure (cat. no. 74104, QIAGEN) with DNase I treatment.

### RNA-seq and analysis

Paired sequence reads underwent quality trimming using Skewer (v.0.2.2) with default settings^58^. Subsequently, RNA-seq reads were aligned to the human reference genome sequence (hg38) using STAR aligner (v.2.5.3a) with default settings^59^. Annotations and genome files (hg38) were obtained from Ensembl (release 105). Uniquely mapped reads with mapping quality (*Q* ≥ 30) were counted across genes with featureCounts (subread 2.0.0)^60^. Using the annotation package org.Hs.eg.db, ribosomal and mitochondrial genes as well as pseudogenes, and genes with no annotation (Entrez Gene ID), were removed before normalization and statistical analysis. In this dataset, only genes with >0.5 counts per million in at least four samples were retained for statistical analysis. Differential gene expression analysis was performed in R (v.3.6.0) with the Bioconductor package edgeR (v.3.36.0)^61^ in RStudio. An FDR < 0.05 using the Benjamini–Hochberg correction^62^ was used.

### Creation of hCOs

hCOs were created as described previously^28,63^. Briefly, day 15 cardiac differentiation was dissociated to a single-cell suspension. Acid-solubilized collagen I (Devro) was salt-balanced with 10× DMEM, pH-neutralized with 0.1 M NaOH and mixed sequentially on ice with Matrigel and cells. Each hCO contained 5 × 10^4^ cells in 2.6 mg ml^−1^ collagen I and 9% Matrigel in a volume of 3.5 µl. After gelling for 60 min at 37 °C and 5% CO_2_, hCOs were cultured in α-MEM GlutaMAX (Thermo Fisher Scientific), 10% FCS, 200 mM l-ascorbic acid 2-phosphate sesquimagnesium salt hydrate (Sigma-Aldrich) and 1% penicillin-streptomycin (Thermo Fisher Scientific) with the medium changed every 2–3 d. Nine days after hCOs were created, active force production was measured by analyzing hCO pole deflection; 10-s videos were collected at 100 Hz using a Leica Thunder DMi8 inverted microscope. Videos were analyzed to describe contractile parameters using a previously described MATLAB code^28^.

### Calcium imaging

Calcium imaging was performed using Fluo-4 AM (cat. no. F-14201, Invitrogen). Briefly, cells were incubated in Tyrode’s buffer (140 mM NaCl, 5.4 mM KCl, 1 mM MgCl_2_, 10 mM glucose, 1.8 mM CaCl_2_, 10 mM HEPES, pH 7.4) containing 5 mM Fluo-4 AM and 0.02% pluronic acid F-127 for 30 min at 37 °C. Cells were then washed with Tyrode’s buffer for 30 min. Line scans were collected at a frequency of 100 Hz using an LSM900 and ×40 oil objective.

### Immunoprecipitation

Lactate-purified CMs were washed with cold PBS before lysis in co-IP buffer (150 mM NaCl, 50 mM Tris-HCl, pH 7.5, 10% glycerol, 0.1% Triton X-100). Lysates were incubated at 4 °C with agitation for 1 h. Insoluble matter was pelleted by centrifugation at 12,000*g* for 15 min at 4 °C. Each reaction was incubated in 2 µg of anti-FLAG M2 (cat. no. F1804, Sigma-Aldrich) for 2 h at 4 °C and incubated with 40 µl washed Dynabeads Protein G (cat. no. 10003D, Invitrogen) for 1 h. Beads were collected using magnetic separation and washed twice in cold lysis buffer followed by three washes in cold PBS. The buffer was aspirated and the beads were snap-frozen on dry ice. For the reverse binding experiments, 2 mg of lysate from metabolically purified ALPK3–SBP3×FLAG hPSC CMs was immunoprecipitated for 3 h at 4 °C using an anti-SQSTM1 antibody (cat. no. ab56416. Abcam) followed by Dynabeads Protein G for 1 h. Beads were washed with co-IP buffer, resuspended in 1× Laemmli sample buffer and heated at 70 °C for 10 min. ALPK3–SBP3×FLAG and SQSTM1 were detected by western blot using anti-FLAG M2 (1:1,000 dilution) or anti-SQSTM1 antibody (1:2,000 dilution), respectively.

### HEK 293 cell transfection

HEK 293FT cells (cat. no. R70007, Invitrogen) were cultured in 1× DMEM, 10% FCS, 0.1 mM nonessential amino acids (Thermo Fisher Scientific), 1% GlutaMAX, 1% penicillin-streptomycin and 500 mg ml^−1^ geneticin (Gibco). HEK 293FT cells were plated at 625,000 cells per well of a six-well culture plate. Plasmids were transfected using Lipofectamine 3000 in the above medium without antibiotics. After 24 h, medium was changed and refreshed to include antibiotics and cells were collected for downstream applications the next day.

### Targeted ALPK3 kinase assay

SQSTM1 containing a C-terminal hemagglutinin (HA) tag was overexpressed in HEK 293FT cells for 72 h using Lipofectamine 3000. Samples were collected in co-IP buffer plus protease inhibitor, snap-frozen on dry ice and stored at −80 °C until use. On the day of the experiment, lysates were thawed on ice and sheared with ten strokes through a 22-gauge needle followed by three strokes through a 27-gauge needle. A BCA assay was performed and 2 mg of lysate was immunoprecipitated for 2 h with a 2 μg of an anti-HA antibody (cat. no. ab9110, Abcam) and Dynabeads Protein A (cat. no. 10002D, Invitrogen) for an additional 1 h. Beads were washed in co-IP buffer before being split equally between tubes. Beads were resuspended in 500 μl of kinase assay buffer plus protease inhibitor. One tube received 5 μg active ALPK3 kinase and one received 5 μg of heat-denatured ALPK3 kinase. Tubes were incubated at 30 °C for 3 h with shaking at 1,200 r.p.m. On completion, beads were washed with kinase assay buffer and split equally again into two tubes. Half were snap-frozen, while half were treated with lambda protein phosphatase (New England Biolabs) before freezing. Samples were thawed and resuspended in 1× Laemmli sample buffer and heated to 70 °C for 10 min. Equal volumes were then run on a 6% acrylamide gel containing 25 μM Phos-tag (Wako Chemicals) to separate phosphorylated species. Blots were transferred to a polyvinylidene fluoride membrane and probed with an anti-phospho-SQSTM1 antibody (T269/S272, 1:1,000 dilution, cat. no. 3121S, Cell Signaling Technology).

### Reporting summary

Further information on research design is available in the Nature Portfolio Reporting Summary linked to this article.

### Supplementary information

Supplementary Information Oligonucleotide sequences for cell mouse line generation.

Reporting Summary

Supplementary Table 1 Mass spectrometry datasets.

Supplementary Video 1 Representative video of beating ALPK3–tdTomato hPSC CMs.

Supplementary Video 2 Three-dimensional reconstruction of confocal Z-stack of live ALPK3–tdTomato stains counterstained with Hoechst 33342.

Supplementary Video 3 High-magnification three-dimensional reconstruction of confocal Z-stack of live ALPK3–tdTomato stains counterstained with Hoechst 33342.

### Source data

Source Data Fig. 1 Unprocessed western blots.

Source Data Fig. 2 Statistical source data.

Source Data Fig. 3 Statistical source data.

Source Data Fig. 4 Statistical source data.

Source Data Fig. 5 Unprocessed western blots.

Source Data Extended Data Fig. 2 Unprocessed western blots.

Source Data Extended Data Fig. 3 Statistical source data.

Source Data Extended Data Fig. 3 Unprocessed western blots.

Source Data Extended Data Fig. 4 Statistical source data.

Source Data Extended Data Fig. 6 Statistical source data.

Source Data Extended Data Fig. 8 Statistical source data.

Source Data Extended Data Fig. 9 Unprocessed western blots.

## Data Availability

All proteomics raw data have been deposited in the PRoteomics IDEntification Database under accession nos. PXD035535 (total and phosphoproteomics data of WT versus *ALPK3*^mut^), PXD035734 (ALPK3–SBP3×FLAG affinity purification) or PXD038544 (global kinase assay). The RNA-seq data have been deposited in the Gene Expression Omnibus under accession no. GSE215304. Other data and reagents are available upon reasonable request. Source data are provided with this paper.
