# Supplementary material for: Alpha kinase 3 signaling at the M-band maintains sarcomere integrity and proteostasis in striated muscle
Source: Nat Cardiovasc Res. 2023 Feb 15;2(2):159–73. doi: 10.1038/s44161-023-00219-9 (PMC11358020; doi:10.1038/s44161-023-00219-9)
Supplement: Supplementary file 1 — Oligonucleotide sequences for cell mouse line generation. [file 44161_2023_219_MOESM1_ESM.pdf]

nature cardiovascular research

Article https://doi.org/10.1038/s44161-023-00219-9

Alpha kinase 3 signaling at the M-band maintains sarcomere integrity and proteostasis in striated muscle

In the format provided by the authors and unedited

Oligonucleotide Sequences

Guide RNA sequences

ALPK3-tdTomato and ALPK3-SBP3XFLAG
5’-GCCCCCAGCCTCTGCGG-3’

$ALPK3^{L639fs/34}$
5’-CCAGGCGCCCGGACACTCA-3’

$ALPK3^{Q1460X}$
5’-GGCCCTGGATGAAGGCAAGC-3’

$ALPK3^{R1792X}$
5’-GATTGCTACCAAACTCCGA-3’

Mouse $Alpk3^{W1538X}$
5’-TCCAGCACTGGCTGTATCAG-3’

Donor repair templates

$ALPK3^{L639fs/34}$
5’-aggcagtcaggcctcttggggaagagggaccccagacTctagtTAGTgggcgcctggggagagtcccaag gggaaggcac-3’

$ALPK3^{Q1460X}$
5’-CGGCGGTCTGGACACAGAGGTGGCCCTGGATGAAGGtAAatAaGAGACACTG GCCAAGCCCAGGAAAGCCAAAGACCTGCTGA-3’

$ALPK3^{R1792X}$
5’-TGACTGGAAGATGACTGATGTGCAGATTGCTACCAAgttgtGAGGGTGAGTGG TTCTTGGGGACAGAATGCCCTCTGGGCGTC-3’

Mouse $Alpk3^{W1538X}$
5’-CCAGCAGCTCCGAGGCCTTGCAGAAATGCCAAACCTTCCAGCACTGGCTGT ATCAGTaGACAAACGGCAGCTTTCTTGTCACAGATCTGACAGgtatgagggtgggggactcacagg tgtg-3’

Screening Primers

ALPK3-tdTomato and ALPK3-SBP3XFLAG
External-F: 5’- CTGGGCCAAGTAGAGAGAC-3’
tdTomato-R: 5’-CTCTTTGATGACCTCCTCGCCCT-3’
External-R: 5’- CTCAAGGATGGTTAGTTGCAGG-3’

$ALPK3^{L639fs/34}$
External-F: 5’-TGGGCACTCCAGACAAGG-3’
Screening-F: 5’-gggaccccagacTctagtTAGT-3’
External-R: 5’-tgtagctggcactccagtcc-3’

$ALPK3^{Q1460X}$
External-F: 5’-GATTTCTCCCTAAGGTCAGAGC-3’
Screening-F: 5’-TGGCCCTGGATGAAGGtAAatAaG-3’
External-R: 5’-gtgcctcctgcctatatctcagg-3’

$ALPK3^{R1792X}$
External-F: 5’-gaagagttttggctctaggaatcc-3’
Screening-F: 5’-GTGCAGATTGCTACCAAgttgt-3’
External-R: 5’-ATCAGTAATAGCTGCCACACC-3’

Mouse Alpk3^{W1538X}

Genotype-F: 5’-ACCAGGGCATGTTCTCTGTGG-3’

Genotype-R: 5’-TGTGGGCTTTGAACTGGACTCC-3’
