# Supplementary material for: Alpha kinase 3 signaling at the M-band maintains sarcomere integrity and proteostasis in striated muscle
Source: Nat Cardiovasc Res. 2023 Feb 15;2(2):159–73. doi: 10.1038/s44161-023-00219-9 (PMC11358020; doi:10.1038/s44161-023-00219-9)
Supplement: Supplementary file 2 — Reporting Summary [file 44161_2023_219_MOESM2_ESM.pdf]

nature portfolio

Corresponding author(s): Enzo R. Porrello and David A. Elliott

Last updated by author(s): Oct 3, 2022

Reporting Summary

Nature Portfolio wishes to improve the reproducibility of the work that we publish. This form provides structure for consistency and transparency in reporting. For further information on Nature Portfolio policies, see our Editorial Policies and the Editorial Policy Checklist.

Statistics

For all statistical analyses, confirm that the following items are present in the figure legend, table legend, main text, or Methods section.

n/a	Confirmed	
☐	☒	The exact sample size (*n*) for each experimental group/condition, given as a discrete number and unit of measurement
☐	☒	A statement on whether measurements were taken from distinct samples or whether the same sample was measured repeatedly
☐	☒	The statistical test(s) used AND whether they are one- or two-sided<br>*Only common tests should be described solely by name; describe more complex techniques in the Methods section.*
☒	☐	A description of all covariates tested
☐	☒	A description of any assumptions or corrections, such as tests of normality and adjustment for multiple comparisons
☐	☒	A full description of the statistical parameters including central tendency (e.g. means) or other basic estimates (e.g. regression coefficient) AND variation (e.g. standard deviation) or associated estimates of uncertainty (e.g. confidence intervals)
☐	☒	For null hypothesis testing, the test statistic (e.g. *F*, *t*, *r*) with confidence intervals, effect sizes, degrees of freedom and *P* value noted<br>*Give P values as exact values whenever suitable.*
☒	☐	For Bayesian analysis, information on the choice of priors and Markov chain Monte Carlo settings
☒	☐	For hierarchical and complex designs, identification of the appropriate level for tests and full reporting of outcomes
☒	☐	Estimates of effect sizes (e.g. Cohen's *d*, Pearson's *r*), indicating how they were calculated

Our web collection on statistics for biologists contains articles on many of the points above.

Software and code

Policy information about availability of computer code

Data collection	Imaging data were collected using ZEISS ZEN 2012 (blue edition) or ZEISS ZEN Black software (version 2.3 SPI) (Zeiss Microscopy, Thornwood, NY) software installed on a ZEISS LSM 780 confocal microscope, LSM 780 confocal microscope or Leica THUNDER Imager. RNA sequencing data were collected with NovaSeq Control Software for the Illumina NovaSeq 6000 sequencing system. Flow cytometry data were collected using the BD LSR Fortessa X-20 Cell Analyser (BD Biosciences, CA) and BD FACSDiva (v9.0.1). Proteomics samples were analysed on a Dionex 3500 nanoHPLC, coupled to an Orbitrap Eclipse mass spectrometer (ThermoFischer Scientific).
Data analysis	Immunofluorescent and calcium images were processed ZEISS ZEN Black software (version 2.3 SPI) (Zeiss Microscopy, Thornwood, NY) and Fiji ImageJ (version: 2.3.0/l.53q). Flow cytometry was analyzed using BD FlowJo (v10.8.1)Human cardiac organoid force analysis was performed using a previously published MATLAB script (Mills, 2017). Proteomics data were analysed using Spectronaut (15.1.210713.50606) and Perseus (Tyanova, S. Nat. Methods 2016). Bulk RNA sequencing analysis was performed using Skewer (v0.2.2) for trimming, aligned with STAR aligner (v2.5.3a). Reads were analysed with featureCounts (subread 2.0.0). Differential gene expression analysis was performed in the R statistical programming language (v3.6.0) with the Bioconductor packages edgeR (v3.36.0).

For manuscripts utilizing custom algorithms or software that are central to the research but not yet described in published literature, software must be made available to editors and reviewers. We strongly encourage code deposition in a community repository (e.g. GitHub). See the Nature Portfolio guidelines for submitting code & software for further information.

Data

Policy information about availability of data

All manuscripts must include a data availability statement. This statement should provide the following information, where applicable:

- Accession codes, unique identifiers, or web links for publicly available datasets
- A description of any restrictions on data availability
- For clinical datasets or third party data, please ensure that the statement adheres to our policy

All proteomics raw data have been deposited in PRIDE: PXD035535 (total and phosphoproteomics data of WT vs. ALPK3mut), or PXD035734 (ALPK3-SBP3XFLAG affinity purification), or PXD038544 (Global kinase assay) and will be made public following acceptance of the manuscript. RNA sequencing has been deposited to the Gene Expression Omnibus: GSE215304 and will be made public following acceptance of the manuscript. Source data are provided with this paper. Other data is available upon reasonable request.

Human research participants

Policy information about studies involving human research participants and Sex and Gender in Research.

Reporting on sex and gender	N/A
Population characteristics	N/A
Recruitment	N/A
Ethics oversight	N/A

Note that full information on the approval of the study protocol must also be provided in the manuscript.

Field-specific reporting

Please select the one below that is the best fit for your research. If you are not sure, read the appropriate sections before making your selection.

☒ Life sciences ☐ Behavioural & social sciences ☐ Ecological, evolutionary & environmental sciences

For a reference copy of the document with all sections, see nature.com/documents/nr-reporting-summary-flat.pdf

Life sciences study design

All studies must disclose on these points even when the disclosure is negative.

Sample size	Sample sizes were based on even group distribution and no statistical method was used to predetermine sample size owing to the exploratory nature of the study. Experiments included a minimum of 3 biological replicates per condition across multiple experiments. Biological replicates were classed as monolayer differentiations. Sample sizes are consistent with best practices in the field.
Data exclusions	Minimal data were excluded from the study. Individual human cardiac organoids were excluded from analyses if they met outlier criteria using Prism (Version 9.3.1) ROUT, Q=1%.
Replication	The results depicted in this manuscript are representative of observations and analyses made across multiple experiments, biological replicates, and technical replicates. Each experiment included a minimum of 3 biological replicates per condition. All replication attempts were successful.
Randomization	No randomization past blinding of experiments was performed in this study. Additional controlling of covariates were not relevant to this study.
Blinding	Proteomics and RNAseq experiments were performed with experimenters blinded to genotype. All mass spectrometry was analyzed blinded to condition. Human cardiac organoid experiments were not blinded due to the automated processes used to collect and analyze results. Where relevant, immunofluorescent images were scored by observers blinded to genotype. All animal studies were performed and analyzed blinded.

Reporting for specific materials, systems and methods

We require information from authors about some types of materials, experimental systems and methods used in many studies. Here, indicate whether each material, system or method listed is relevant to your study. If you are not sure if a list item applies to your research, read the appropriate section before selecting a response.

Materials & experimental systems

n/a	Involved in the study	
☐	☒	Antibodies
☐	☒	Eukaryotic cell lines
☒	☐	Palaeontology and archaeology
☐	☒	Animals and other organisms
☒	☐	Clinical data
☒	☐	Dual use research of concern

Methods

n/a	Involved in the study	
☒	☐	ChIP-seq
☐	☒	Flow cytometry
☒	☐	MRI-based neuroimaging

Antibodies

Antibodies used

Anti-Sarcomeric Alpha Actinin antibody Abcam (ab68167) 1 in100 flow cytometry; 1:1000 immunofluorescence; 1:5000 Western Blot
Anti-Sarcomeric Alpha Actinin antibody Sigma-Aldrich (A7811) 1 in100 flow cytometry; 1:1000 immunofluoresence; 1:5000 Western Blot
Anti-MYOMI antibody Abeam (ab205618) 1 in 200 immunofluorescence
Anti-OBSCN antibody Abeam (ab121652) 1 in 500 immunofluorescence
Anti-SQSTMI / p62 antibody Abeam (ab56416) 1 in 100 immunofluorescence ; 1 in 2,000 Western Blotting
Anti-FLAG antibody Sigma-Aldrich (F1804) 1 in 300 immunofluorescence; 1 in 1,000 Western Blotting
Anti-FLAG antibody Cell Signalling Technologies (14793) 1 in 500 immunofluoresence
PE/Cy7 anti-human CD90 antibody Biolegend (328124) 1 in 100 flow cytometry
Anti-Cardiac Troponin T antibody Abeam (ab8295) 1 in 100 flow cytometry
Anti-MYBPC3 (E7) antibody Santa Cruz Biotechnologies (sc-137180) 1 in 300 immunofluorescence; 1 in 5,000 Western Blotting
Anti-phosphoSQSTM1/p62 (Thr269/272) antibody Cell Signalling Technologies (13121) 1 in 1,000 Western Blotting
Anti-SQSTM1/p62 antibody Sigma-Aldrich (P0067) 1 in 500 immunofluorescence

Validation

Antibodies were previously validated for immunofluorescence of human or mouse tissue either in the laboratories of ourselves or commercial suppliers.

Eukaryotic cell lines

Policy information about cell lines and Sex and Gender in Research

Cell line source(s)

All hPSC cell lines were derivatives of the female HES3 NKX-GFP reporter cell line (Elliott et al. Nature Methods 2011 Oct 23;8(12)). The HES3 cell line is available from WiCell (ES03), and was originally reported in Richards et al. Nature Biotechnology 2002 Sep;20(9):933-6.

HEK293FT were sourced from Invitrogen (Cat# R70007)

Authentication

hPSC lines are checked for pluripotency and genomic integrity by immunofluorescence of pluripotency markers and molecular karyotyping. HEK293FT were not authenticated.

Mycoplasma contamination

All cell lines confirmed negative for mycoplasma.

Commonly misidentified lines
(See ICLAC register)

No commonly misidentified cell lines were used.

Animals and other research organisms

Policy information about studies involving animals; ARRIVE guidelines recommended for reporting animal research, and Sex and Gender in Research

Laboratory animals

3-8 week-old C57BL/6J Mice edited to contain a variant (W1538X) in the ALPK3 gene, were used in this study. Mice were fed standard chow and water ad libitum, and maintained in a 12-hour light/12-hour dark cycle at ambient room temperature.

Wild animals

No wild animals were used in this study

Reporting on sex

Echocardiography experiments contained a mixed gender.
WT: n=9 (3M, 6F)
Het: n=8 (4M,4F)
Hom: n=9 (5M, 4F)

Field-collected samples

No field collected samples were used in this study.

Ethics oversight

All experiments were approved by the Animal Ethics Committee of the Murdoch Children's Research Institute, Parkville, Australia

Note that full information on the approval of the study protocol must also be provided in the manuscript.

Flow Cytometry

Plots

Confirm that:

☒ The axis labels state the marker and fluorochrome used (e.g. CD4-FITC).

☒ The axis scales are clearly visible. Include numbers along axes only for bottom left plot of group (a 'group' is an analysis of identical markers).

☒ All plots are contour plots with outliers or pseudocolor plots.

☒ A numerical value for number of cells or percentage (with statistics) is provided.

Methodology

Sample preparation	Flow cytometry of reporter line-derived hPSC-cardiomyocytes using endogenous fluorescence was performed following Trypsin-EDTA (0.25%), phenol red (ThermoFisher Scientific) for 5 minutes followed by inactivation with aMEM +10% FBS. Cell suspensions were filtered with 100um strainer to produce a single cell suspension and resuspended in PBS/2% FBS. For intracellular flow, cells were fixed in 2% paraformaldehyde (PFA) for 536 10 minutes prior to permeabilization with 0.25% triton X-100. Primary antibody staining used alpha-actinin (Sigma-Aldrich A7811 (Abcam ab11370) or cardiac troponin-T (ab8295). For surface staining of stromal cell populations, a PE-Cy7 conjugated anti-CD90 antibody was stained in PBS/2% FBS for 30 minutes in the dark at room temperature. Following staining, cells were washed 3 times with PBS/2% FBS and resuspended in PBS/2% FBS containing either DAPI or propidium iodide.
Instrument	Flow cytometry was performed using the BD LSR Fortessa X-20 Cell Analyzer (BD Biosciences, California, U.S.A.).
Software	Data acquisition and analysis was performed using FACsDiva versions 8.0.1 and 9.0.1 (BD Biosciences) and FlowJo Software.
Cell population abundance	Cardiac myocyte abundance was assessed using alpha-actinin (Sigma-Aldrich A7811 (Abcam ab11370) or cardiac troponin-T (ab8295) and ranged between ~70-90%.
Gating strategy	FSC/SSC analysis was initially performed to identify individual live cells and exclude cell debris and clusters of two or more cells. Isotype control antibodies were used to determine appropriate gating strategies for myocyte and stromal cell markers. For endogenous reporter-based flow cytometry (ALPK3-tdTomato), the parental hPSC line which does not contain the tdTomato fluorescent reporter was used for gating.

☒ Tick this box to confirm that a figure exemplifying the gating strategy is provided in the Supplementary Information.
