# Supplementary figures and images for: Alpha kinase 3 signaling at the M-band maintains sarcomere integrity and proteostasis in striated muscle
Source: Nat Cardiovasc Res. 2023 Feb 15;2(2):159–73. doi: 10.1038/s44161-023-00219-9 (PMC11358020; doi:10.1038/s44161-023-00219-9)

Uncropped WB images for Figure 1F

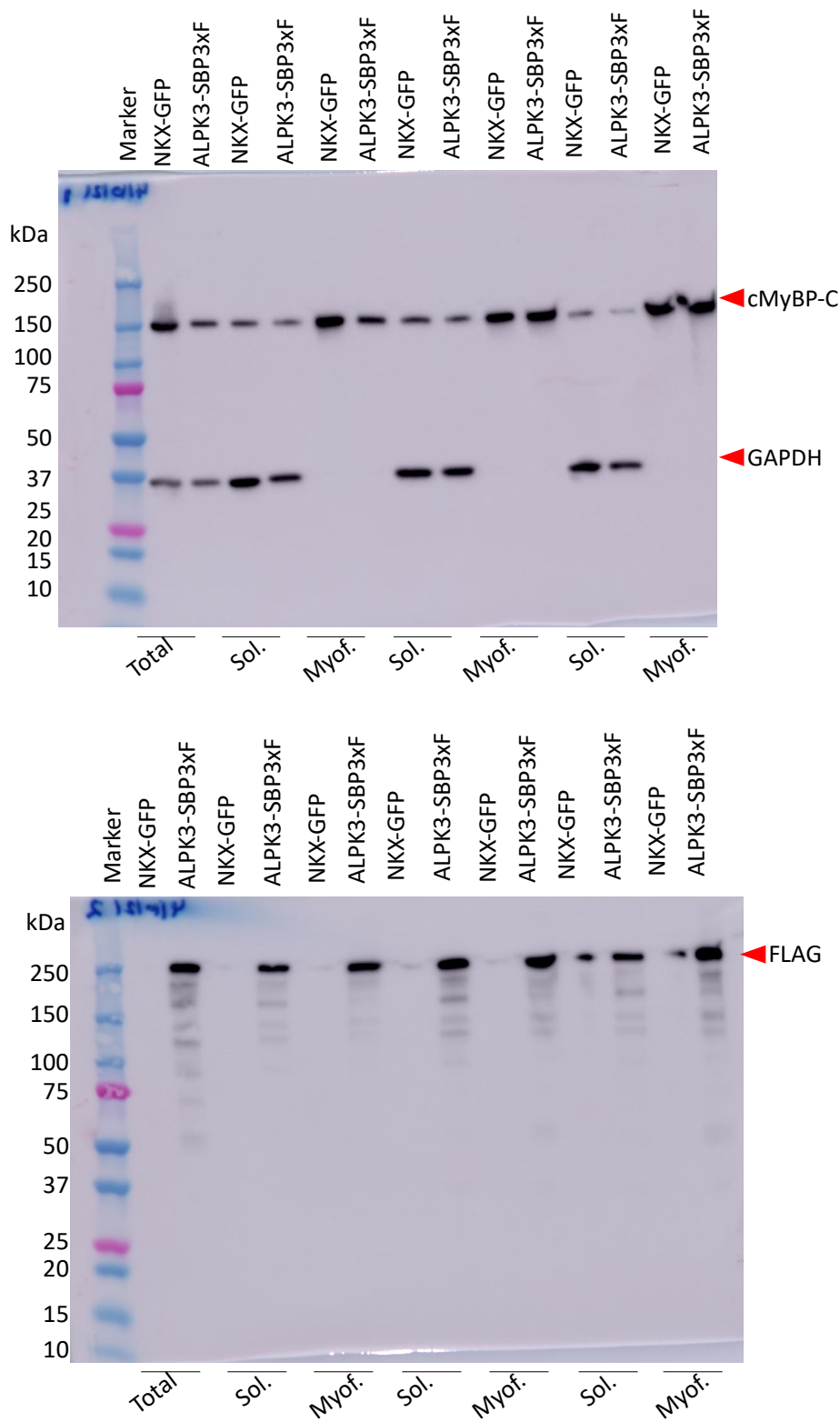

Supplement: Supplementary file 7 — Unprocessed western blots. [file 44161_2023_219_MOESM7_ESM.pdf]

Uncropped WB Images for Figure 5

Figure 5D

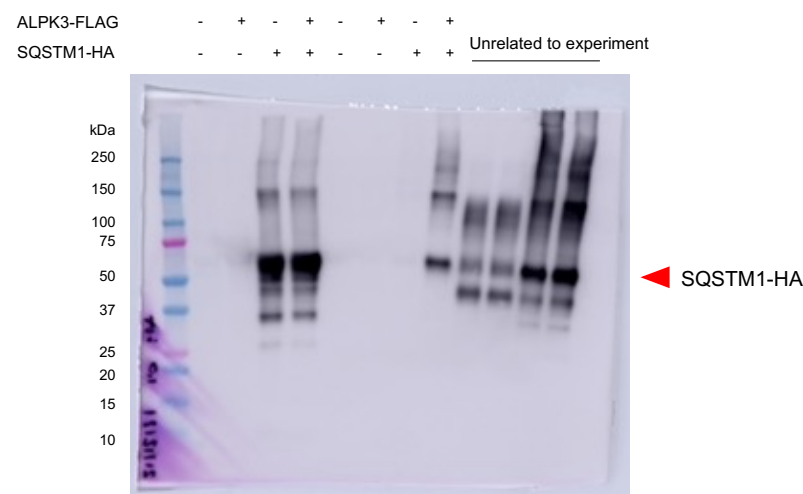

Figure 5F

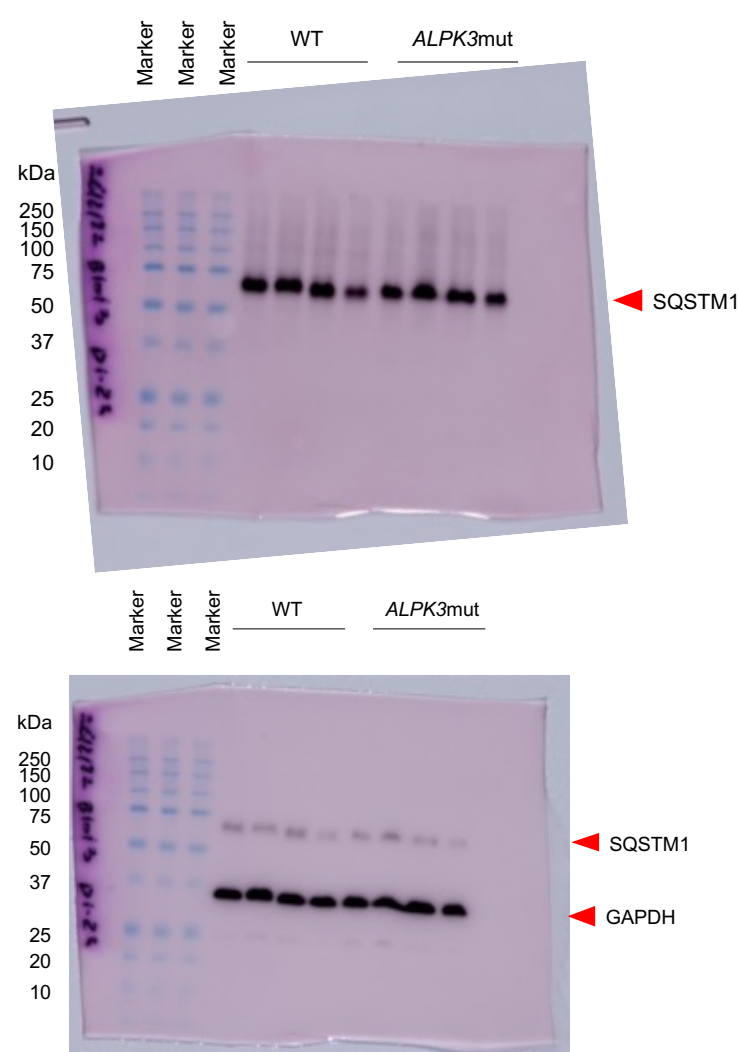

Supplement: Supplementary file 11 — Unprocessed western blots. [file 44161_2023_219_MOESM11_ESM.pdf]

Uncropped WB Images for Extended Data Figure 3

Extended Data Figure 3G

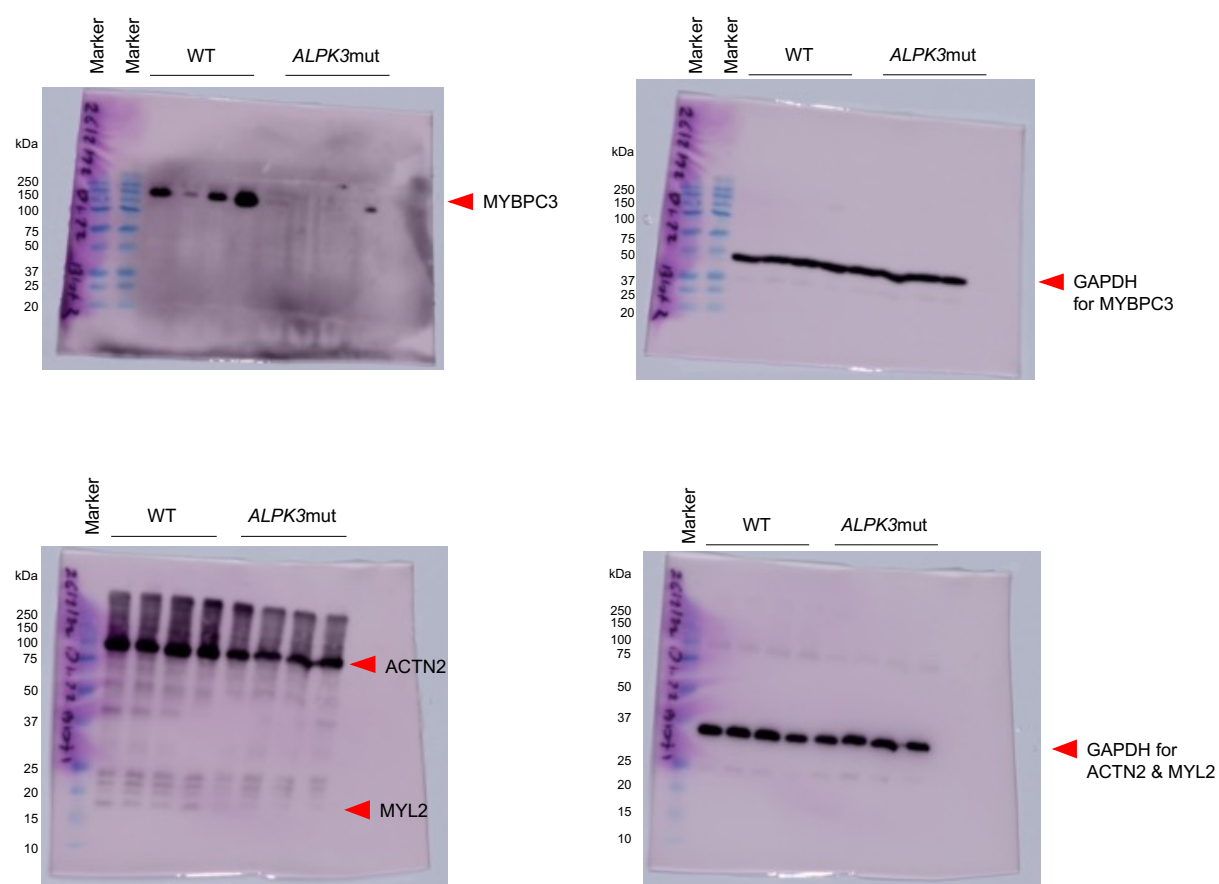

Supplement: Supplementary file 14 — Unprocessed western blots. [file 44161_2023_219_MOESM14_ESM.pdf]
