# Supplementary material for: Alpha kinase 3 signaling at the M-band maintains sarcomere integrity and proteostasis in striated muscle
Source: Nat Cardiovasc Res. 2023 Feb 15;2(2):159–73. doi: 10.1038/s44161-023-00219-9 (PMC11358020; doi:10.1038/s44161-023-00219-9)
Supplement: Supplementary file 18 — Unprocessed western blots. [file 44161_2023_219_MOESM18_ESM.pdf]

Uncropped Western Blots for Extended Data Figure 9

Extended Data Figure 9B

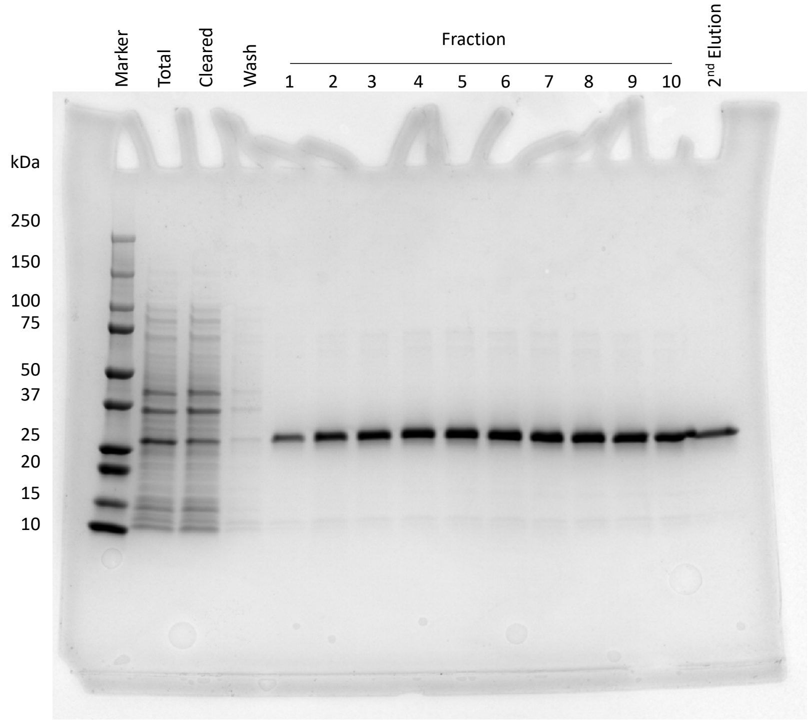

Extended Data Figure 9C

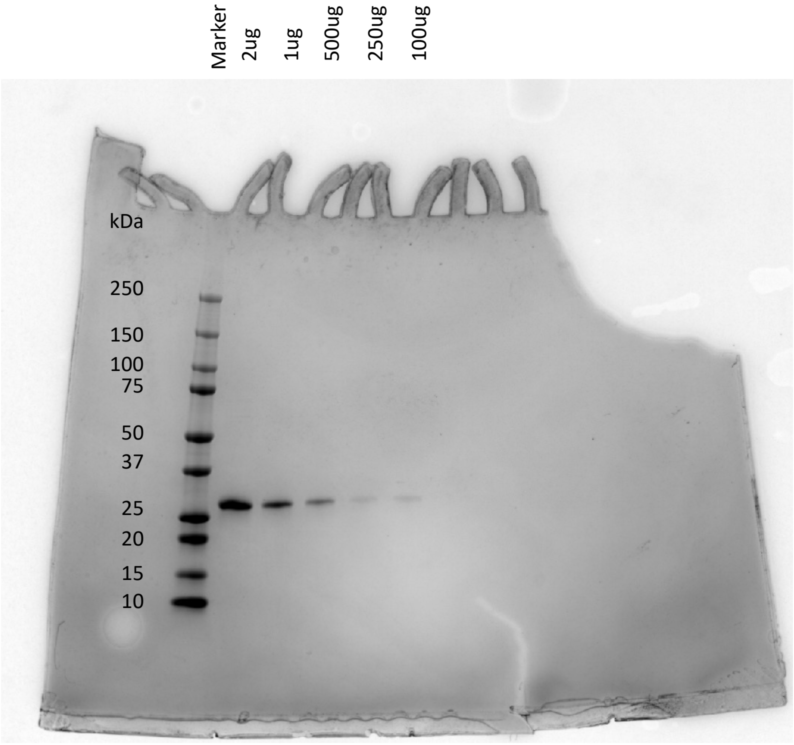

Extended Data Figure 9E

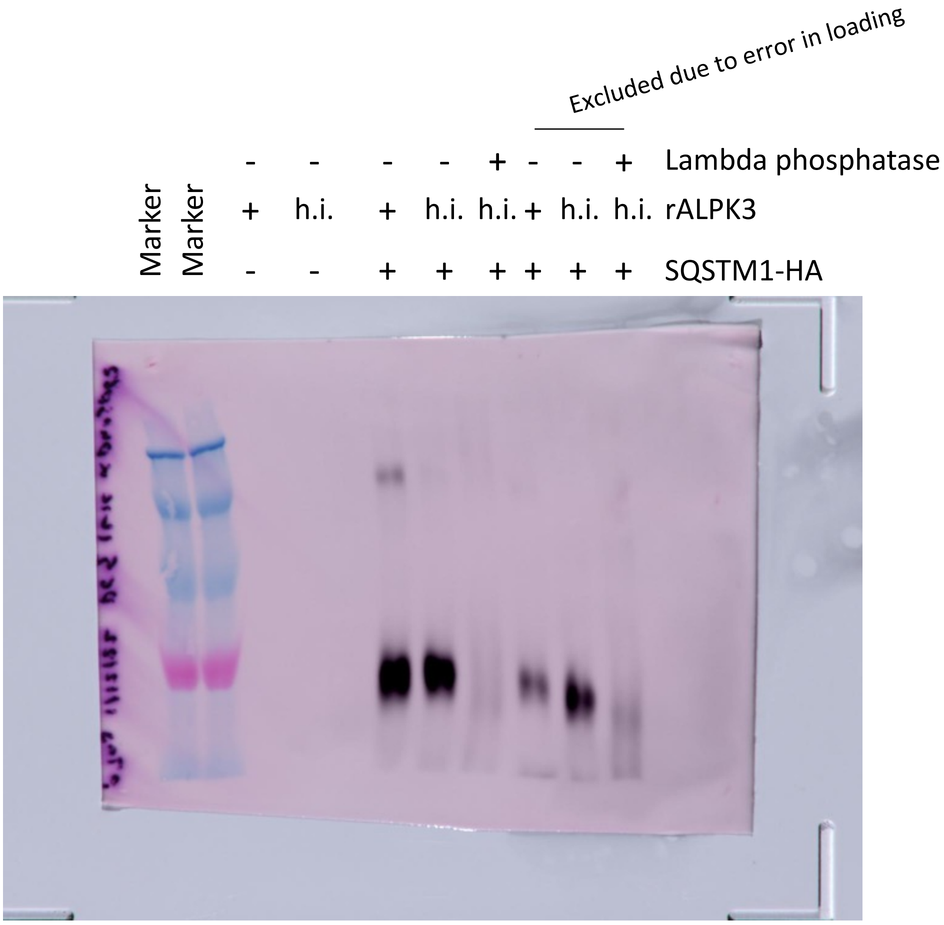

Extended Data Figure 9F

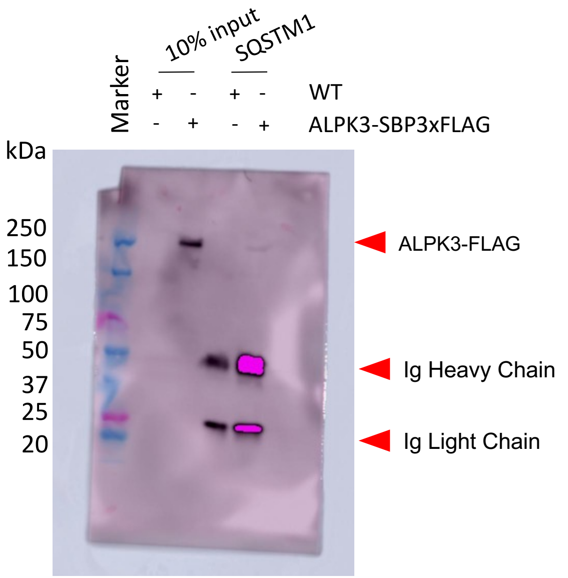

CoIP: HA
WB: Anti-FLAG

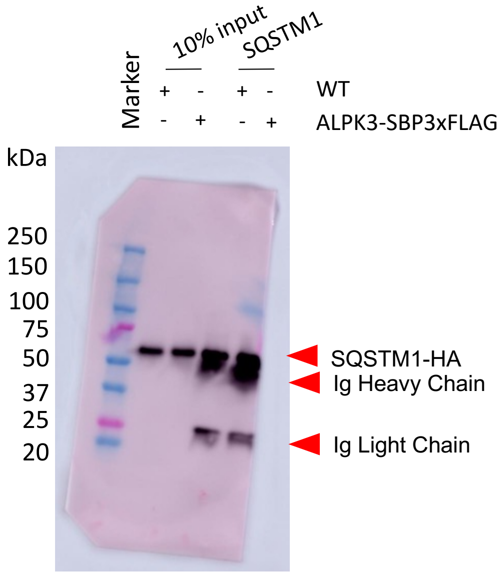

CoIP: HA
WB: Anti-HA
