# Supplementary material for: Alpha kinase 3 signaling at the M-band maintains sarcomere integrity and proteostasis in striated muscle
Source: Nat Cardiovasc Res. 2023 Feb 15;2(2):159–73. doi: 10.1038/s44161-023-00219-9 (PMC11358020; doi:10.1038/s44161-023-00219-9)

Uncropped WB images for Extended Data Figure 2

Extended Data Figure 2E

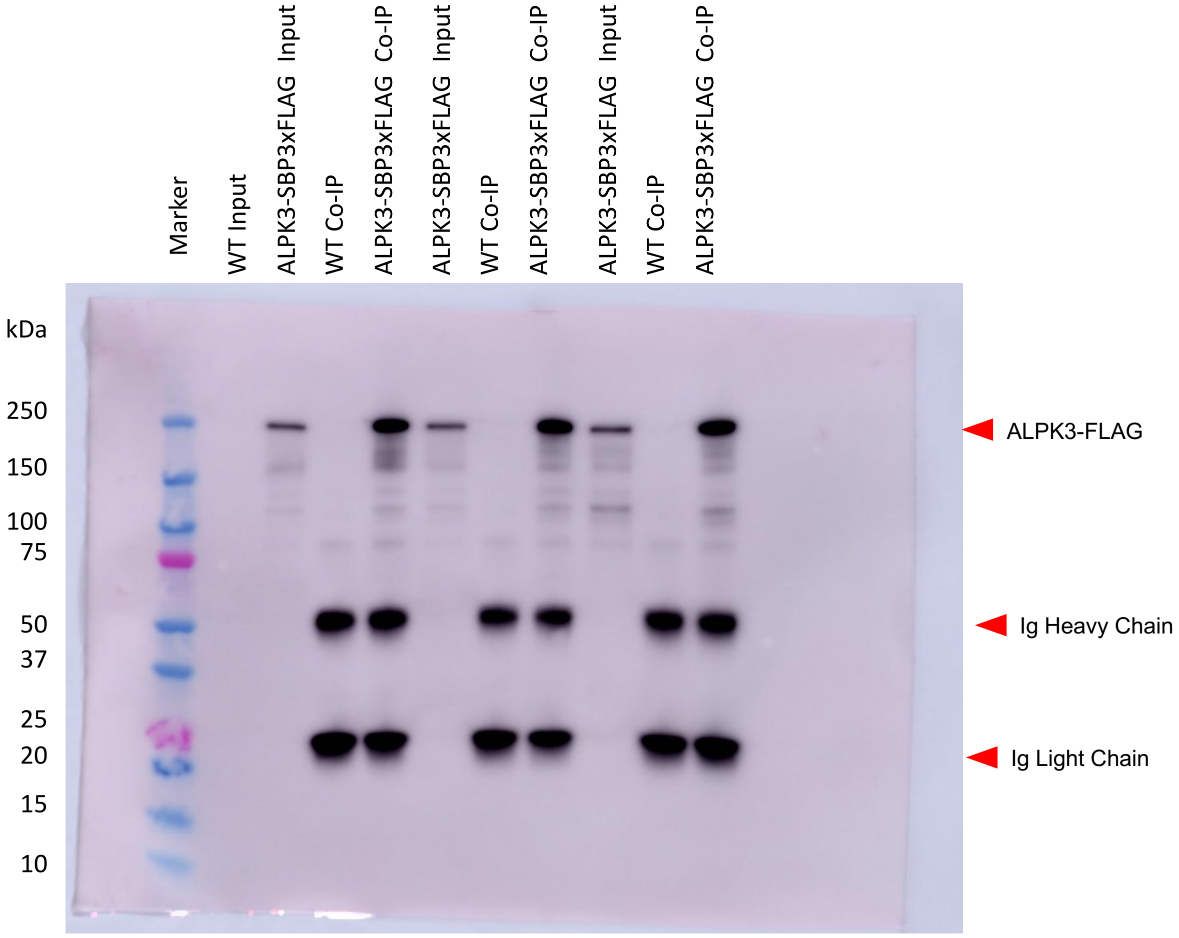

Supplement: Supplementary file 12 — Unprocessed western blots. [file 44161_2023_219_MOESM12_ESM.pdf]
